# Detection and Growth Pattern of Arcuate Fasciculus from Newborn to Adult

**DOI:** 10.3389/fnins.2017.00389

**Published:** 2017-07-14

**Authors:** Molly Wilkinson, Ashley R. Lim, Andrew H. Cohen, Albert M. Galaburda, Emi Takahashi

**Affiliations:** ^1^Department of Behavioral Neuroscience, Northeastern University Boston, MA, United States; ^2^Division of Newborn Medicine, Department of Medicine, Boston Children's Hospital, Harvard Medical School Boston, MA, United States; ^3^Department of Psychology, Northeastern University Boston, MA, United States; ^4^Department of Neurology, Beth Israel Deaconess Medical Center, Harvard Medical School Boston, MA, United States

**Keywords:** development, arcuate fasciculus, hemispheric asymmetry, human, diffusion MRI, tractography

## Abstract

Fractional anisotropy (FA) threshold is commonly used to perform diffusion MRI tractography. However, FA threshold may be one aspect of tractography that needs additional scrutiny in accurately assessing pathways in immature, developing brains, as well as in adult brains. Using high-angular resolution diffusion MRI (HARDI) tractography without an FA threshold, we identified the arcuate fasciculus (AF) of 83 healthy subjects ranging in age from 40 gestational weeks (GW) (newborns) to 28-year-old adults. The AF was identified in both hemispheres in all subjects with high inter-rater reliability. The detected AF included regions with very low FA values. The entire AF was segmented into anterior, posterior, and long tracts. Growth and laterality patterns were investigated using tract count (number of detected streamlines), total volume of imaging voxels (touched by the detected streamlines), mean length, mean FA, and mean apparent diffusion coefficient (ADC). Comparison of subjects under 3 years old, to those that were older, revealed the three AF tracts that took different developmental courses. As expected, the anterior and long tracts showed lower ADC values in subjects over 3 years old, while the posterior tract showed higher ADC in that same age range. The posterior tract did not show age-related effect in terms of FA, tract count, length, and volume. These results suggest that the posterior AF tract shows a matured state, indexed by most of the used measurements in early postnatal developmental ages, and ADC is a measurement that can detect further maturation of the posterior tract. Interestingly, in all tracts, hemispheric asymmetries were found in raw (left<right), and in whole brain (WB)-normalized (left>right) tract count, as well as in raw volume (left<right). In raw, and in WB-normalized length, as well as in WB-normalized volume, rightward asymmetry (left<right) was found only in the anterior tract; other tracts were not significantly affected by hemisphere. Although many previous studies have observed a leftward asymmetry in the AF, rightward asymmetry has also been reported in other studies, and together with the present report, the results in the literature are likely to reflect differences in the methods used.

## Introduction

Hemispheric cerebral asymmetry is an important accompaniment of many cognitive functions, especially language processing (Balsamo et al., 2002; Coney, 2002; Zatorre et al., 2002; Vernooij et al., 2007; Matsumoto et al., 2008; Holtgraves and Felton, 2011; Sun et al., 2015). The left hemisphere dominance for most language functions is widely accepted, and is implemented in Broca's and Wernicke's areas of the brain and connected by the arcuate fasciculus (AF) (Geschwind, 1970; Catani et al., 2005), among other regions. The AF, a subdivision of the superior longitudinal fasciculus (SLF), consists of three branches: the long, main branch, which connects Broca's area and Wernicke's area (Geschwind, 1970; Catani et al., 2005); the anterior, fronto-parietal branch, which connects the inferior parietal lobule to Broca's area; and the posterior, temporo-parietal branch, which connects the inferior parietal lobule to Wernicke's area (Catani and Mesulam, 2008).

Hemispheric asymmetries in the arcuate and the superior longitudinal fasciculi have been found using structural MR imaging and diffusion tensor imaging (DTI) tractography, though these studies mostly focused on adolescent and adult brains (Büchel et al., 2004; Liu et al., 2010; Thiebaut de Schotten et al., 2011a,b; Häberling et al., 2013). Thus, fetal and infant AF research has not received the same amount of attention as adolescent and adult AF research. This is because imaging during these stages of development poses major challenges. Dubois et al. (2009) imaged infants between 1 and 4 months of age by using diffusion tractography to examine the early port-natal development of the AF. The temporal component did not show significant hemispheric asymmetry, whereas the parietal component showed leftward asymmetry. Based on the work by Chi et al. (1977), Dubois et al. (2009) proposed that the lack of asymmetry in the temporal component could be attributed to the right sulci developing slightly before the left, implying the left side would continue to mature and ultimately become asymmetric. Liu et al. (2010) studied preterm babies, born between 26 and 34 GW, scanning them between 35 and 42 GW, and were able to identify the developing tracts of the SLF. They found volume and microstructural leftward asymmetries in the parieto-temporal part of the SLF and no asymmetry in the fronto-parietal part of the SLF, linked to the AF. In this study a “tract” refers to a bundle of fibers (e.g., each segment of AF), while “pathways” are the tractography fibers found inside of these tracts.

Another important aspect of imaging the AF, while using diffusion tractography in infant brains, is the general use of fractional anisotropy (FA) thresholds to terminate fiber tracking in brain regions with low FA values, which allows for the visualization of the pathways as they end in the gray matter. Since much of the immature white matter in the developing brain has low FA values, it is important to consider not using an FA threshold for tractography in infants. This issue may also apply to adults, as FA values tend to decrease toward the gray-white junction.

Previous studies have reported a difference in end location of the AF at different developmental stages. Diffusion MR tractography showed the tract ending in the premotor cortex in infants, caudal to the termination in the inferior frontal gyrus (prefrontal) among adults (Perani et al., 2011; Brauer et al., 2013). However, Dubois' most recent study (Dubois et al., 2016) investigated the development of the AF in infants between the ages of 6 and 22 weeks and did not confirm these findings. They suggested that the previous report could have resulted from technical limitations in young brains. Using similar arguments, Yeatman et al. (2011) discussed possible reasons why so many researchers have not been able to identify the AF on the right side of the brain. Thus, they used a probabilistic tracking algorithm, instead of a deterministic one, to assist with the identification of the right AF. Noise and interference from the tracts of the nearby SLF helped contribute to difficulties in identifying the right AF.

Using DTI, many studies found leftward structural asymmetry in the AF of adults (Nucifora et al., 2005; Parker et al., 2005; Vernooij et al., 2007; Upadhyay et al., 2008; Propper et al., 2010; Takao et al., 2011; Vandermosten et al., 2013) and children 5 years old and older (Lebel and Beaulieu, 2009; Sreedharan et al., 2015). A few studies (e.g., Uda et al., 2015) investigated white matter pathways from infant to adult ages looking at growth curves of pathways, but without reporting on hemispheric asymmetry. Therefore, it is still not clear whether younger newborns and toddlers have an established leftward laterality in the AF or not (Song et al., 2014).

Utilizing information of water diffusivity in many directions, high-angular resolution diffusion MR imaging (HARDI) tractography can help distinguish multiple separate pathways as they cross within an imaging voxel (e.g., Tuch et al., 2003), even in immature brains (e.g., Takahashi et al., 2012, 2014) where, compared to adults, there is a surplus of unmyelinated or fewer myelinated fibers. This technique could potentially provide advantages over traditional DTI (Frank, 2002; Tournier et al., 2004, 2008), which typically only allows for the detection of one direction of water diffusivity per voxel.

The human brain grows substantially during the first few years of life (Thompson, 2001; Lippé et al., 2009; Gredebäck and Kochukhova, 2010; Berchicci et al., 2011; Bellagamba et al., 2015) and reaches close to its adult size by the age of 3 years old. Likewise, gray and white matter achieves adult-like MRI contrast (Holland et al., 1986; Barkovich et al., 1988; Barkovich and Kjos, 1988; Martin et al., 1988; Hayakawa et al., 1990), diffusion properties (e.g., Cohen et al., 2016), and some psychological milestones (e.g., Schimdt and Beauchamp, 1988; Berthier et al., 2000; Whiten et al., 2006; Keen and Shutts, 2007; Keitel et al., 2013; Smith et al., 2015). Therefore, in the current study, we focused on the differences between two groups: 3 year-olds and under, and those over 3 years of age. We characterized the normal developmental neuroanatomy of the AF from the newborn period to adult ages using HARDI tractography with no FA threshold. We did not use FA thresholds in order to maximize the extent of the AF included in our measurements. Our previous study, that ranged from early gestational fetuses to 3-year-old toddlers, found no significant asymmetry of the AF (Song et al., 2014), while other studies suggest that there is leftward hemispheric asymmetry in AF before the age of 3 (Dubois et al., 2009). In the current study, we increased the number of subjects under 3 years old compared to our previous study, but similar to our previous study, we applied a new tractography approach without the use of FA thresholds. We hypothesized that sub-segments (anterior, posterior, and long segments) of the AF would develop differentially.

## Materials and methods

### Subjects and MR imaging acquisition

This is a retrospective study of clinical MRI data, and the Institutional Review Board at Boston Children's Hospital deemed this an exempt project waiving the need for further consent to be obtained because the research is retrospective only involves existing data with no risk to patient confidentiality.

All living participants had clinically-indicated brain MRI studies that were diagnostically interpreted to show no abnormalities. In newborns, indications for imaging included concern for hypoxic ischemic injury, apnea and transient choreiform movements after an upper respiratory tract infection. None had clinical concerns for a congenital malformation or genetic disorder. In children and adult subjects, common reasons for brain MRI studies included headaches and infections in ear/nose/throat and sleeping problems.

The following inclusion criteria were used: (1) subject age at time of exam must have been below 30 years old, (2) MRI exams must have been performed on a 3T scanner and included T1W, T2W, as well as our standard diffusion weighted imaging sequence with 30 directions (described below), (3) MRI examinations must have been reported as normal. Exclusion criteria were as follows: (1) any reported MRI abnormality, (2) motion or other artifacts noted in the MRI report or on image inspection in consensus of two of the authors of this paper.

For 83 reportedly healthy subjects, from newborns to 28 years of age (Y) (eighteen 0–1 Y, ten 2–4 Y, fourteen 5–7 Y, eleven 8–10 Y, twelve 11–14 Y, nine 15–17 Y, nine 18–28 Y; 43 females and 40 males), we performed T1-weighted MPRAGE imaging, T2-weighted turbo spin-echo imaging, and isotropic diffusion-weighted spin-echo echo-planar imaging. Thirty diffusion-weighted measurements (*b* = 1,000 s/mm^2^) and five non-diffusion-weighted measurements (*b* = 0 s/mm^2^) were acquired on a 3T MR system (Skyra, Siemens Medical Systems, Erlangen. Germany) with *TR* = 10 s; *TE* = 88 ms; δ = 12.0 ms; Δ = 24.2 ms; spatial resolution 2mm isotropic; matrix size = 128 × 128, iPAT = 2.

### Diffusion data reconstruction for tractography

DiffusionToolkit and TrackVis (trackvis.org) were used to reconstruct and visualize tractography pathways. Tractography pathways were reconstructed using a HARDI (Q-ball) model with a streamline/FACT algorithm and a 45° angle threshold. Brain mask volumes were used to terminate tractography structures instead of the standard fractional anisotropy (FA) threshold (Schmahmann et al., 2007; Wedeen et al., 2008; Takahashi et al., 2010, 2011, 2012, 2014; Song et al., 2014) because progressive myelination and crossing fibers in the developing brain can result in low FA values that may potentially incorrectly terminate tractography tracing in brain regions with low FA values.

### Tract delineation

**Figure 1:**
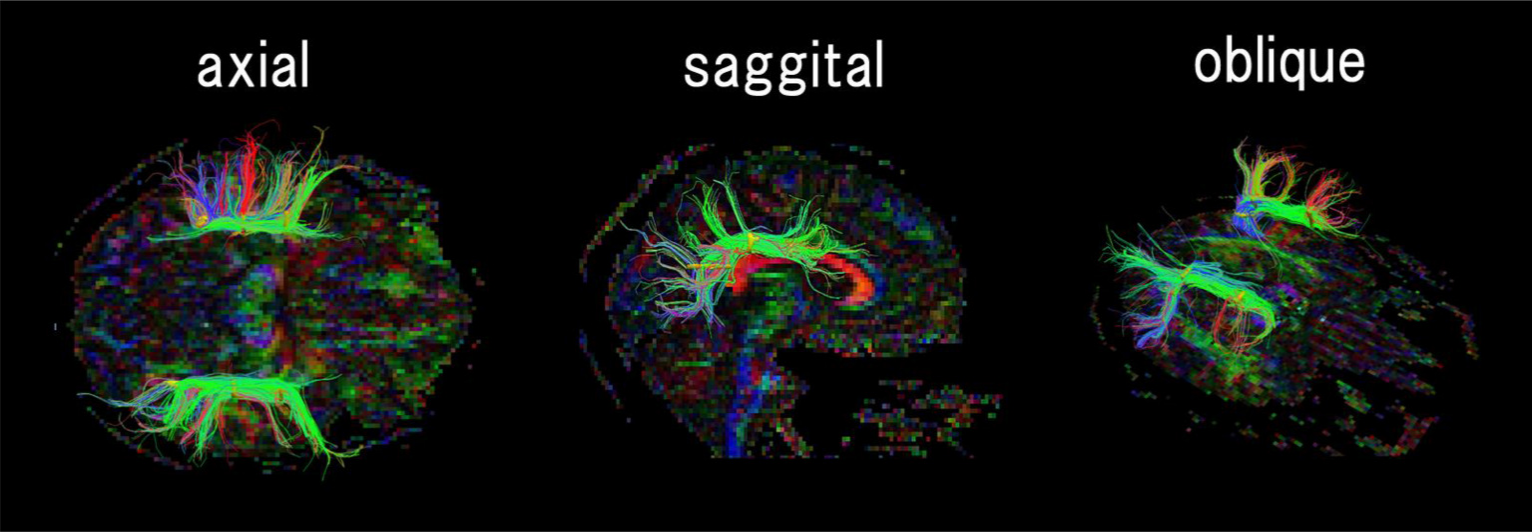
An example of regions of interest (ROIs, shown in yellow) placement for identifying the arcuate fasciculus. According to the human brain fiber atlas (Catani and Thiebaut de Schotten, 2008), ROIs were carefully placed along expected AF fiber pathways on a FA color map. The color coding of fibers is based on a standard RGB code, applied to the vector between the end-points of each fiber (Red: left-right, Green: anterior-posterior, and Blue: dorsal-ventral).

**Figure 2:**
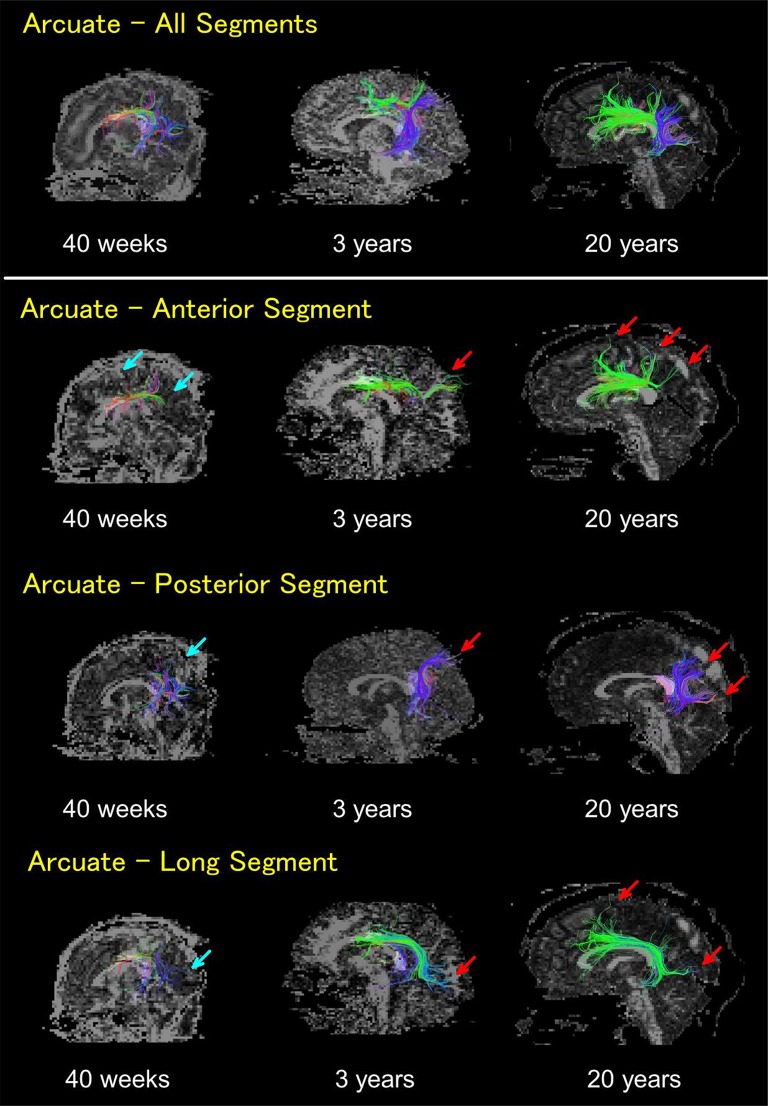
HARDI tractography pathways on 40 gestational weeks newborn, 3 years old, and 20 years old subjects. Whole arcuate fasciculus pathways (top row), anterior segments (second row), posterior segments (third row), and long segments (bottom row). In newborns, the anterior, posterior, and long segments of the AF were all thin and sparse, with short pathways running into the cortex that were not detected in regions close to the brain surface (light blue arrows). By 3 years old, such pathways running into the cortex became obvious, with their numbers increasing until adult ages (red arrows). The color coding of fibers is based on a standard RGB code, applied to the vector between the end-points of each fiber (Red: left-right, Green: anterior-posterior, and Blue: dorsal-ventral).

Anatomic and tractography atlases (Catani and Thiebaut de Schotten, 2008; Mori and Tournier, 2013) were used to guide regions of interest (ROIs) placements on non-diffusion-weighted (b0) images and color FA maps in order to delineate the pathways of interest (Figure 1). We first carefully identified the whole AF by placing hand-drawn ROIs continuously along white matter regions shown in the atlases above (Figure 2, top row). Then, three segments were separated using additional ROIs. For the anterior segment (Figure 2, second row), we placed two ROIs at the edge of the dorsal anterior and dorsal posterior parts of the AF, and identified pathways running through both ROIs. For the posterior segment (Figure 2, third row), two ROIs were placed at the edges of the posterior dorsal and posterior ventral AF regions, and pathways running through the two ROIs were separated. Finally, for the long segment (Figure 2, bottom row), a ROI was placed in the middle part of the fasciculus running in the dorsal anterior-posterior direction, and the other was placed in the posterior edge of the fasciculus. The sizes and locations of all the ROIs were carefully optimized so as to not include other white matter pathways, as well as to not miss real arcuate pathways. We occasionally used additional ROIs to exclude clearly different pathways from the AF pathways that were difficult to separate from the arcuate pathways by only using the ROIs described above (e.g., cingulate pathways vs. the long segment of the AF in a few subjects).

For each subject, a “whole-brain” tract group was created to sum all of the streamlines identified within the entire intra-cranial brain space. These whole brain pathways were used for the whole-brain normalization described below.

### Quantification

We measured mean FA, mean ADC, tract number, mean length, and total volume of detected streamlines per segmented tract in each subject. We used TrackVis to calculate these measures. Raw data of each type of measurement were further normalized using two schemes: an “18Y+” normalization and a “whole-brain” (WB) normalization. In the 18Y+ normalization, individual subject data was divided by the average value in all subjects 18 years or older, separately in the left and right hemispheres. The purpose of this normalization was to compare an individual's tracts to those of a mature adult. We only showed scatter plots of 18+ normalized data, as the raw and 18+ normalized data had identical statistical analysis results. In WB normalization, each individual's quantities from the left and right hemispheres were divided by the sum of all intracranially-identified brain pathways in the same individual (i.e., the WB tractgroup described above). The purpose of this normalization was to account for individual variations in brain volume and tract characteristics.

### Statistical analysis

Subject data was divided into two groups: those 3 years and younger (24 subjects) and those over 3 years of age (59 subjects). All statistical analyses were performed with SPSS version 19.0 (IBM SPSS, Chicago, IL, USA). Statistical significance was set at *p* < 0.05 (Bonferroni corrected).

### Inter-rater reliability

To assess inter-rater reliability, the data from 6 subjects (3 newborns and 3 adults) were re-analyzed by a newly trained researcher, using the same ROI approach. Originally identified AF pathways were re-identified, and Tract Count, Volume, Length, FA, and ADC values were extracted. “Tract count” is a term in TrackVis that indicates the number of tractography fibers. For each measure (Tract Count etc.), values from all identified pathways in 6 subjects were tested for correlation across the 2 individual ways of pathway identification. Inter-rater reliabilities were: for Tract Count, 93.5%, for Volume, 95.1%, for Length, 86.9%, for FA, 82.6%, and for ADC, 83.3%.

## Results

### Qualitative descriptions

The anterior, posterior, and long segments of the AF in newborns were all thin and sparse with short pathways running into the cortex that were not detected in regions close to the cerebral surface (Figure 2, light blue arrows). By 3 years of age, pathways running into the cortex became obvious, with their numbers increasing until adult ages (Figure 2, red arrows). Multiple tractography views of pathways from representative subjects at different ages can be found in Supplementary Figures S1–S4.

Scatter plots from the raw and 18+ normalized data (Supplementary Figures S5, S6) showed moderate increases in tract count, volume, length, and FA values, and decrease in ADC values by age. FA and ADC values seemed to reach a plateau around 2 or 3 years old. Scatter plots for the WB normalized data (Supplementary Figure S7) showed gradual decreases in tract count, volume, length, and ADC values by age.

### Quantitative analyses

#### Raw data

**Figure 3:**
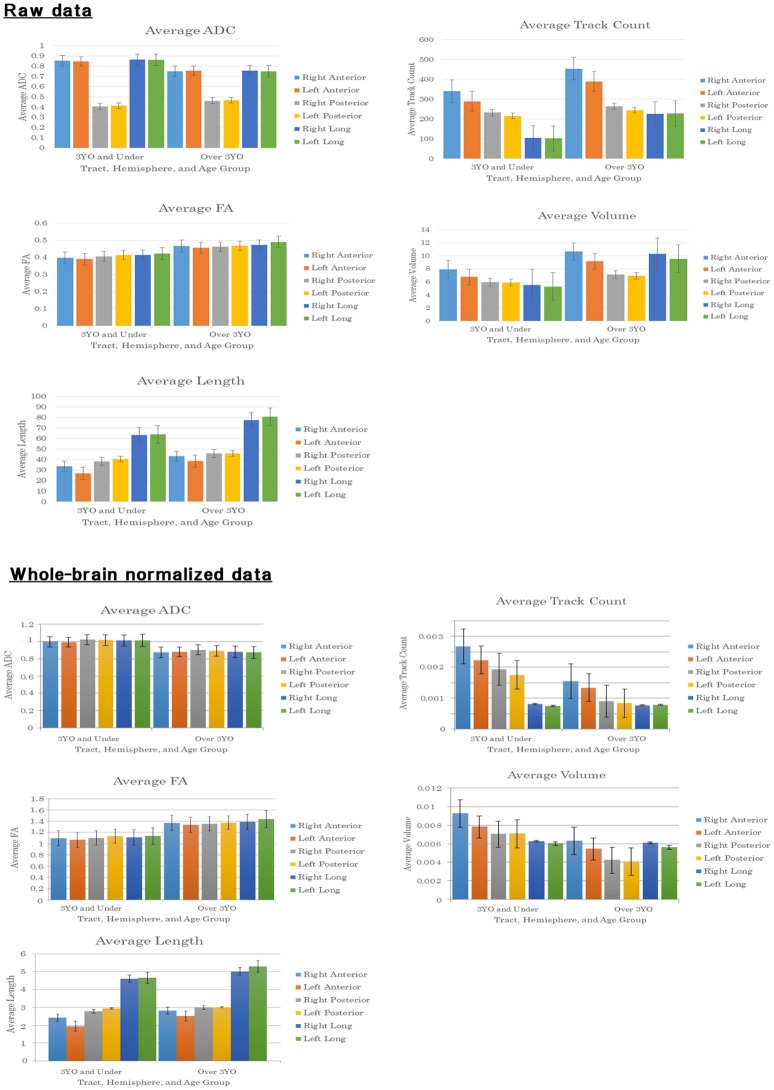
Bar graphs of averaged values and standard deviations for each measure studied: ADC (mm^2^/s), volume (ml) and length (mm).

A three-way ANOVA was conducted to examine the effects of between-subjects factor age (3 years and under, over 3 years) and within-subjects factors hemisphere (left, right) and tract (anterior, long, posterior) on raw data for ADC, FA, length, tract count, and volume (Figure 3, Raw data).

The analysis of age, hemisphere, and tract effects on ADC measurement revealed a significant main effect of age [*F*_(1, 470)_ = 21.540, *p* < 0.005, η_*p*_^2^ = 0.044] indicating that subjects over the age of 3 had higher ADC values compared to those 3 and under. Subjects over 3 had an average ADC value of 0.155, whereas those 3 and under showed an average of 0.141. There was no significant main effect of hemisphere [*F*_(1, 470)_ = 0.454, *p* = 0.501, η_*p*_^2^ = 0.001]. The left AF had an average ADC value of 0.153, while the right AF had an average of 0.150. The main effect of tract [*F*_(2, 470)_ = 5442.747, *p* < 0.005, η_*p*_^2^ = 0.959] indicates that posterior tracts have significantly higher ADC values in comparison with anterior and long tracts (*p* < 0.005; *p* < 0.005). There were no significant two-way interactions between age and hemisphere [*F*_(1, 470)_ = 0.009, *p* = 0.925, η_*p*_^2^ < 0.005] or between tract and hemisphere [*F*_(2, 470)_ = 0.460, *p* = 0.631, η_*p*_^2^ = 0.002]. The two-way interaction between age and tract [*F*_(2, 470)_ = 22.035, *p* < 0.005, η_*p*_^2^ = 0.086] and simple main effects testing suggested that the ADC of anterior, long, and posterior tracts were affected by age. Anterior tracts displayed a significant interaction with age [*F*_(1, 158)_ = 13.631, *p* < 0.005, η_*p*_^2^ = 0.079] indicating that the anterior tracts of the AF in subjects 3 and under had a higher ADC value compared to those over 3. Anterior tracts in subjects 3 and under had an average ADC value of 0.00085, while anterior tracts in subjects older than 3 had an average of 0.00075. Long tracts displayed a significant interaction with age [*F*_(1, 154)_ = 14.251, *p* < 0.005, η_*p*_^2^ = 0.085] indicating that the long tracts of the AF in subjects 3 and under had higher ADC values in comparison with those over 3. Long tracts in subjects 3 and under were an average ADC value of 0.00086, while it was an average of 0.00075 in subjects over 3. Posterior tracts displayed a significant interaction with age [*F*_(1, 158)_ = 22.384, *p* < 0.005, η_*p*_^2^ = 0.124] indicating that subjects over 3 had higher ADC values compared to those 3 and under. Subjects over 3 had an average ADC value for the posterior tracts of 0.46478, while those 3 and under had an average posterior tract value of 0.40937. There was no three-way interaction found among age, hemisphere, and tract [*F*_(2, 470)_ = 0.009, *p* = 0.991, η_*p*_^2^ < 0.005].

The analysis of age, hemisphere, and tract effects on FA measurement revealed a significant main effect of age [*F*_(1, 470)_ = 923.036, *p* < 0.005, η_*p*_^2^ = 0.165] indicating that subjects over 3 had higher FA values in comparison to those 3 and under. Subjects over 3 had an average FA value of 0.466, while those 3 and under showed an average of 0.406. There was no significant main effect of hemisphere [*F*_(1, 470)_ = 0.419, *p* = 0.518, η_*p*_^2^ = 0.001]. The left AF had an average FA value of 0.454, while the right AF had an average of 0.449. The main effect of tract [*F*_(2, 470)_ = 4.040, *p* = 0.018, η_*p*_^2^ = 0.017] indicates that long tracts have significantly higher FA values than anterior tracts (*p* = 0.004). Long tracts had an average of 0.464 and anterior tracts had an average FA value of 0.442. Posterior tracts had an average of 0.449. There were no significant two-way interactions between age and hemisphere [*F*_(1, 470)_ = 0.010, *p* = 0.922, η_*p*_^2^ < 0.005], age and tract [*F*_(2, 470)_ = 0.311, *p* = 0.733, η_*p*_^2^ = 0.001], and tract and hemisphere [*F*_(2, 470)_ = 1.077, *p* = 0.342, η_*p*_^2^ = 0.004]. There was no three-way interaction found among age, hemisphere, and tract [*F*_(2, 470)_ = 0.109, *p* = 0.897, η_*p*_^2^ < 0.005].

The analysis of age, hemisphere, and tract effects on length revealed a significant main effect of age [*F*_(1, 470)_ = 117.206, *p* < 0.005, η_*p*_^2^ = 0.200] indicating that subjects over 3 had longer tracts as compared to subjects 3 and under. Subjects over 3 had an average length of 55.23 mm, while subjects 3 and under showed an average of 43.89 mm. There was no significant main effect of hemisphere [*F*_(1, 470)_ = 0.808, *p* = 0.369, η_*p*_^2^ = 0.002]. The left whole AF had an average length of 51.72, while the right whole AF had an average of 52.43. The main effect of tract [*F*_(2, 470)_ = 473.349, *p* < 0.005, η_*p*_^2^ = 0.668] suggests that long tracts were significantly longer than anterior and posterior tracts (*p* < 0.005; *p* < 0.005). Long tracts had an average length of 74.91, while anterior tracts had an average of 37.87 and posterior tracts had an average of 44.02. There was no significant two-way interaction between age and hemisphere [*F*_(1, 470)_ = 0.195, *p* = 0.659, η_*p*_^2^ < 0.005]. The two-way interaction between age and tract [*F*_(2, 470)_ = 6.772, *p* = 0.001, η_*p*_^2^ = 0.028] and simple main effects testing indicated that the length of anterior, long, and posterior tracts were affected by age. Anterior tracts displayed a significant interaction with age [*F*_(1, 158)_ = 51.133, *p* < 0.005, η_*p*_^2^ = 0.244] indicating that the anterior tracts of the AF in subjects over 3 were longer than those 3 and under. Anterior tracts in subjects over 3 had an average length of 40.87, while anterior tracts in subjects 3 and under had an average of 30.30. Long tracts displayed a significant interaction with age [*F*_(1, 154)_ = 49.621, *p* < 0.005, η_*p*_^2^ = 0.244] indicating that the long tracts of the AF in subjects older than 3 were longer than in those 3 and under. Subjects older than 3 had an average length of 78.99, while subjects 3 and under had an average of 63.63. Posterior tracts displayed a significant interaction with age [*F*_(1, 158)_ = 19.242, *p* < 0.005, η_*p*_^2^ = 0.109] indicating that they were longer than in subjects over 3 compared to those 3 and under. Tracts in subjects over 3 had an average length of 45.82, while those 3 and under had an average of 39.47. The two-way interaction between hemisphere and tract [*F*_(2, 470)_ = 5.673, *p* = 0.004, η_*p*_^2^ = 0.024] and the simple main effects testing indicated that the length of anterior tracts was affected by hemisphere. Anterior tracts displayed a significant interaction with hemisphere [*F*_(1, 158)_ = 14.308, *p* < 0.005, η_*p*_^2^ = 0.083] indicating that the anterior tract of the AF in the right hemisphere were longer than those in the left. Anterior tracts in the right hemisphere had an average length of 40.45, while anterior tracts in the left had an average of 35.29. Long tracts did not have a significant interaction with hemisphere [*F*_(1, 154)_ = 0.692, *p* = 0.407, η_*p*_^2^ = 0.004]. The long tracts of the AF in the right hemisphere had an average length of 73.69 and tracts of the left had an average of 76.13. Posterior tracts of the AF did not display a significant interaction with hemisphere [*F*_(1, 158)_ = 0.573 *p* = 0.450, η_*p*_^2^ = 0.004]. Right hemisphere AF posterior tracts had an average length of 43.67 and those of the left had an average of 44.35. There was no three-way interaction found among age, hemisphere, and tract [*F*_(2, 470)_ = 0.543, *p* = 0.582, η_*p*_^2^ = 0.002].

The analysis of age, hemisphere, and tract effects on tract count revealed a significant main effect of age [*F*_(1, 470)_ = 55.107, *p* < 0.005, η_*p*_^2^ = 0.105] indicating that subjects over 3 had higher tract count than those 3 and under. Subjects over 3 had an average tract count of 301.12, while those 3 and under showed an average of 217.51. The main effect of hemisphere [*F*_(1, 470)_ = 4.660, *p* = 0.031, η_*p*_^2^ = 0.010] shows that tracts in the right hemisphere had significantly higher tract counts than those in the left. The tracts in the right hemisphere had an average tract count of 291.00, while those on the left side had an average of 264.75. The main effect of tract [*F*_(2, 470)_ = 101.580, *p* < 0.005, η_*p*_^2^ = 0.302] indicated that anterior tracts had significantly higher tract counts than long and posterior tracts (*p* < 0.005; *p* < 0.005) and that posterior tracts had significantly higher tract counts than long tracts (*p* < 0.005). Anterior tracts had an average of 391.13; posterior tracts had an average of 245.65, while long tracts had an average of 194.79. There was no significant two-way interaction between age and hemisphere [*F*_(1, 470)_ = 0.024, *p* = 0.877, η_*p*_^2^ < 0.005]. The two-way interaction between age and tract [*F*_(2, 470)_ = 6.165, *p* = 0.002, η_*p*_^2^ = 0.009] and simple main effects testing indicated that the tract count of anterior and long tracts were affected by age. Anterior tracts displayed a significant interaction with age [*F*_(1, 158)_ = 21.732, *p* < 0.005, η_*p*_^2^ = 0.121] indicating that the anterior tracts of the AF in subjects over 3 had higher tract counts compared to those 3 and under. Anterior tracts in subjects over 3 had an average tract count of 421.48, while anterior tracts in subjects 3 and under had an average of 314.59 Long tracts displayed a significant interaction with age [*F*_(1, 154)_ = 51.047, *p* < 0.005, η_*p*_^2^ = 0.249] indicating that the long tracts of the AF in subjects older than 3 had higher tract counts than those 3 and under. Subjects older than 3 had an average tract count of 227.78, while subjects 3 and under had an average of 103.69. Posterior tracts did not have a significant interaction with age [*F*_(1, 158)_ = 2.241, *p* = 0.136, η_*p*_^2^ = 0.014]. Subjects over 3 had an average tract count of 254.10, while those 3 and under had an average of 224.35. There was no two-way interaction between hemisphere and tract [*F*_(2, 470)_ = 2.114, *p* = 0.122, η_*p*_^2^ = 0.009]. There was no three-way interaction found among age, hemisphere, and tract [*F*_(2, 470)_ = 0.047, *p* = 0.954, η_*p*_^2^ < 0.005].

The analysis of age, hemisphere, and tract effects on volume showed a significant main effect of age [*F*_(1, 470)_ = 98.788, *p* < 0.005, η_*p*_^2^ = 0.174] indicating that subjects over 3 had higher volumes of the whole AF than those 3 and under. Subjects over the age of 3 had an average volume of 8.96 ml, while subjects 3 and under showed an average of 6.26 ml. The main effect of hemisphere [*F*_(1, 470)_ = 5.646, *p* = 0.018, η_*p*_^2^ = 0.012] shows that AF tracts in the right hemisphere had significantly higher volumes than those in the left. The tracts in the right hemisphere had an average volume of 8.58 ml, while those on the left side had an average of 7.85 ml. The main effect of tract [*F*_(2, 470)_ = 20.918, *p* < 0.005, η_*p*_^2^ = 0.082] indicates that anterior and long tracts had significantly higher volumes than posterior tracts (*p* < 0.005; *p* < 0.005). Anterior tracts had an average volume of 9.18 ml and long tracts had an average of 8.73 ml while posterior tracts had an average of 6.73 ml. There was no significant two-way interaction between age and hemisphere [*F*_(1, 470)_ = 0.408, *p* = 0.523, η_*p*_^2^ = 0.001]. The two-way interaction between age and tract [*F*_(2, 470)_ = 12.707, *p* < 0.005, η_*p*_^2^ = 0.051] and simple main effects testing indicated that the volume of anterior, long, and posterior tracts were affected by age. Anterior tracts displayed a significant interaction with age [*F*_(1, 158)_ = 28.803, *p* < 0.005, η_*p*_^2^ = 0.154], indicating that the anterior tracts of the AF in subjects over 3 had higher volumes than those 3 years and under. Anterior tracts in subjects over 3 had an average volume of 9.91 ml, while it was an average of 7.36 for subjects 3 and under. Long tracts displayed a significant interaction with age [*F*_(1, 154)_ = 73.387, *p* < 0.005, η_*p*_^2^ = 0.323] indicating that the long tracts of the AF in subjects older than 3 had higher volumes than those 3 and under. Subjects older than 3 had an average volume of 9.93 ml whereas subjects 3 and under had an average of 5.42 ml. Posterior tracts displayed a significant interaction with age [*F*_(1, 158)_ = 7.021, *p* = 0.009, η_*p*_^2^ = 0.043] indicating that the posterior tracts of the AF in subjects older than 3 had higher volumes compared to subjects 3 and under. Subjects over the age of 3 had an average volume of 7.05 ml, while those 3 and under had an average of 5.93 ml. There was no two-way interaction between hemisphere and tract [*F*_(2, 470)_ = 12.707, *p* = 0.177, η_*p*_^2^ = 0.007]. There was no three-way interaction found among age, hemisphere, and tract [*F*_(2, 470)_ = 0.034, *p* = 0.967, η_*p*_^2^ < 0.005].

#### Whole-brain (WB) normalized data

A three-way ANOVA was conducted to examine the effects of between-subjects factor age (3 years and under, over 3 years), and within-subjects factors hemisphere (left, right) and tract (anterior, long, posterior) on whole-brain normalized data for ADC, FA, length, tract count, and volume (Figure 3, Whole-brain normalized data).

The analysis of age, hemisphere, and tract effects on ADC measurement did not show a significant main effect of age [*F*_(1, 437)_ = 2.564, *p* = 0.110, η_*p*_^2^ = 0.006]. Subjects over 3 had an average whole-brain normalized ADC value of 182.39, while subjects 3 and under showed an average of 174.87. There was no significant main effect of hemisphere [*F*_(1, 437)_ = 0.123, *p* = 0.726, η_*p*_^2^ < 0.005]. The left AF had an average whole-brain normalized ADC value of 181.95, while the right AF had an average of 179.14. The main effect of tract [*F*_(2, 437)_ = 1,796.037, *p* < 0.005, η_*p*_^2^ = 0.892] indicates that posterior tracts have significantly higher whole-brain normalized ADC values than anterior and long tracts (*p* < 0.005; *p* < 0.005). Posterior tracts had an average ADC value of 535.09, while anterior tracts averaged at 0.91 and long tracts with 0.91.There were no significant two-way interactions between age and hemisphere [*F*_(1, 437)_ = 0.002, *p* = 0.964, η_*p*_^2^ < 0.005], age and tract [*F*_(1, 437)_ = 2.647, *p* = 0.072, η_*p*_^2^ < 0.012], and tract and hemisphere [*F*_(2, 437)_ = 0.124, *p* = 0.883, η_*p*_^2^ = 0.001]. There was no three-way interaction found among age, hemisphere, and tract [*F*_(2, 437)_ = 0.002, *p* = 0.998, η_*p*_^2^ < 0.005].

The analysis of age, hemisphere, and tract effects on whole-brain normalized FA revealed a significant main effect of age [*F*_(1, 437)_ = 337.236, *p* < 0.005, η_*p*_^2^ = 0.436] indicating that subjects over 3 had higher FA values than those 3 and under. Subjects over 3 had an average FA value of 1.37, while subjects 3 years and under showed an average of 1.13. There was no significant main effect of hemisphere [*F*_(1, 437)_ = 0.646, *p* = 0.422, η_*p*_^2^ = 0.001]. The left AF had an average FA value of 1.31, while the right AF had an average of 1.30. The main effect of tract [*F*_(2, 437)_ = 5.338, *p* = 0.005, η_*p*_^2^ = 0.024] indicates that long tracts have significantly higher FA values than anterior and posterior tracts (*p* < 0.005; *p* = 0.005). Long tracts had an average of 1.34, while anterior tracts had an average FA value of 1.28 and posterior had an average of 1.30. There were no significant two-way interactions between age and hemisphere [*F*_(1, 437)_ = 0.006, *p* = 0.940, η_*p*_^2^ < 0.005], age and tract [*F*_(2, 437)_ = 0.689, *p* = 0.502, η_*p*_^2^ = 0.003], and tract and hemisphere [*F*_(2, 470)_ = 2.964, *p* = 0.053, η_*p*_^2^ = 0.013]. There was no three-way interaction found among age, hemisphere, and tract [*F*_(2, 437)_ = 0.130, *p* = 0.878, η_*p*_^2^ = 0.001].

The analysis of age, hemisphere, and tract effects on whole-brain normalized length revealed a significant main effect of age [*F*_(1, 437)_ = 27.228, *p* < 0.005, η_*p*_^2^ = 0.059] indicating that subjects over 3 had longer tract length than those 3 and under. Subjects over 3 had an average length of 3.63, while 3 and under showed an average of 3.22. There was no significant main effect of hemisphere [*F*_(1, 437)_ = 0.867, *p* = 0.352, η_*p*_^2^ = 0.002]. The left AF had an average length of 3.49, while the right AF had an average of 3.54. The main effect of tract [*F*_(2, 437)_ = 455.441, *p* < 0.005, η_*p*_^2^ = 0.676] indicates that long tracts were significantly longer than posterior and anterior tracts (*p* < 0.005; *p* < 0.005) and posterior tracts significantly longer than anterior tracts (*p* < 0.005). Long tracts had an average length of 5.08, while posterior tracts had an average of 2.98 and anterior tracts had an average of 2.54. There was no significant two-way interaction between age and hemisphere [*F*_(1, 437)_ = 0.276, *p* = 0.600, η_*p*_^2^ = 0.001]. The two-way interaction between age and tract [*F*_(2, 437)_ = 4.372, *p* = 0.013, η_*p*_^2^ = 0.020] and simple main effects testing indicated that the length of anterior and long tracts were affected by age. Anterior tracts displayed a significant interaction with age [*F*_(1, 147)_ = 27.246, *p* < 0.005, η_*p*_^2^ = 0.156] indicating that the anterior tracts of the AF in subjects over 3 were longer than those 3 and under. Anterior tracts in subjects over 3 had an average length of 2.67, while anterior tracts in subjects 3 and under had an average of 2.19. Long tracts displayed a significant interaction with age [*F*_(1, 143)_ = 11.001, *p* = 0.001, η_*p*_^2^ = 0.071] indicating that the long tracts of the AF in subjects older than 3 were longer than those 3 and under. Subjects older than 3 had an average length of 5.22, while subjects 3 and under had an average of 4.67. Posterior tracts did not display a significant interaction with age [*F*_(1, 147)_ = 0.603, *p* = 0.439, η_*p*_^2^ = 0.004]. Subjects over 3 had an average length of 3.00, while those 3 and under had an average of 2.92. The two-way interaction between hemisphere and tract [*F*_(2, 437)_ = 5.854, *p* = 0.003, η_*p*_^2^ = 0.026] and simple main effects testing indicated that the length of anterior tracts were affected by hemisphere. Anterior tracts displayed a significant interaction with hemisphere [*F*_(1, 147)_ = 19.777, *p* < 0.005, η_*p*_^2^ = 0.119] indicating that the anterior tracts of the AF in the right hemisphere were longer than those in the left. Anterior tracts in the right hemisphere had an average length of 2.72, while anterior tracts in the left had an average of 2.35. Long tracts did not have a significant interaction with hemisphere [*F*_(1, 143)_ = 0.302, *p* = 0.583, η_*p*_^2^ = 0.002]. The long tracts of the right hemisphere had an average length of 5.00 and tracts of the left had an average of 5.16. Posterior tracts did not display a significant interaction with hemisphere [*F*_(1, 147)_ = 1.344, *p* = 0.248, η_*p*_^2^ = 0.009]. Right hemisphere posterior tracts had an average length of 2.94 and those of the left had an average of 3.02. There was no three-way interaction found among age, hemisphere, and tract [*F*_(2, 437)_ = 0.828, *p* = 0.438, η_*p*_^2^ = 0.004].

The analysis of age, hemisphere, and tract effects on whole-brain normalized tract count revealed a significant main effect of age [*F*_(1, 437)_ = 108.144, *p* < 0.005, η_*p*_^2^ = 0.198] indicating that subjects 3 and under had a higher tract count than those older than 3. Subjects 3 under had an average tract count of 0.0016, while subjects older than 3 showed an average of 0.0010. The main effect of hemisphere [*F*_(1, 437)_ = 6.413, *p* = 0.012, η_*p*_^2^ = 0.014] shows that tracts in the left hemisphere had significantly higher tract counts than those in the right. The tracts in the left hemisphere had an average tract count of 0.0011, while those on the right had an average of 0.0013. The main effect of tract [*F*_(2, 437)_ = 148.856, *p* < 0.005, η_*p*_^2^ = 0.405] indicates that anterior tracts had significantly higher tract counts than long and posterior tracts (*p* < 0.005; *p* < 0.005) and that posterior tracts had significantly higher tract counts than long tracts (*p* < 0.005). Anterior tracts had an average of 0.0017, long tracts had an average of 0.0008, and posterior tracts had an average of 0.0011. There was no significant two-way interaction between age and hemisphere [*F*_(1, 437)_ = 0.606, *p* = 0.437, η_*p*_^2^ = 0.001]. The two-way interaction between age and tract [*F*_(2, 437)_ = 34.398, *p* < 0.005, η_*p*_^2^ = 0.136] and simple main effects testing indicated that the tract count of anterior tracts was affected by age. Anterior tracts displayed a significant interaction with age [*F*_(1, 147)_ = 75.873, *p* < 0.005, η_*p*_^2^ = 0.340] indicating that the anterior tracts of the AF in subjects 3 and under had higher tract counts than those older than 3. Anterior tracts in subjects 3 and under had an average tract count of 0.0023, while anterior tracts in subjects over 3 had an average of 0.0014. Long tracts did not display a significant interaction with age [*F*_(1, 143)_ = 1.306, *p* = 0.255, η_*p*_^2^ = 0.009], Subjects 3 and under had an average tract count of 0.0007, while subjects older than 3 had an average of 0.0008. Posterior tracts did not have a significant interaction with age [*F*_(1, 147)_ = 72.550, *p* < 0.005, η_*p*_^2^ = 0.330]. Subjects 3 and under had an average tract count of 0.0018, while those older than 3 had an average of 0.0009. There was no two-way interaction between hemisphere and tract [*F*_(2, 437)_ = 2.182, *p* = 0.114, η_*p*_^2^ = 0.010]. There was no three-way interaction found among age, hemisphere, and tract [*F*_(2, 437)_ = 0.032, *p* = 0.968, η_*p*_^2^ < 0.005].

The analysis of age, hemisphere, and tract effects on whole-brain normalized volume revealed a significant main effect of age [*F*_(1, 437)_ = 81.554, *p* < 0.005, η_*p*_^2^ = 0.157] indicating that subjects 3 and under had higher volumes compared to those older than 3. Subjects 3 and under had an average volume of 0.007, while those older than 3 showed an average of 0.006. The main effect of hemisphere [*F*_(1, 437)_ = 5.327, *p* = 0.021, η_*p*_^2^ = 0.012] shows that tracts in the right hemisphere had significantly higher volumes than those in the left. The tracts in the right hemisphere had an average volume of 0.0061, while tracts on the left side had an average of 0.0056. The main effect of tract [*F*_(2, 437)_ = 20.899, *p* < 0.005, η_*p*_^2^ = 0.087] indicates that anterior tracts had significantly higher volumes than long and posterior tracts (*p* = 0.004; *p* < 0.005) and long tracts had significantly higher volumes than posterior (*p* < 0.005). Anterior tracts had an average volume of 0.0066, long tracts had an average of 0.0059, and posterior tracts had an average of 0.0051. There was no significant two-way interaction between age and hemisphere [*F*_(1, 437)_ = 0.015, *p* = 0.901, η_*p*_^2^ < 0.005]. The two-way interaction between age and tract [*F*_(2, 437)_ = 19.037, *p* < 0.005, η_*p*_^2^ = 0.080] and simple main effects testing indicated that the volume of anterior and posterior tracts were affected by age. Anterior tracts displayed a significant interaction with age [*F*_(1, 147)_ = 50.150, *p* < 0.005, η_*p*_^2^ = 0.254] indicating that the anterior tracts of the AF in subjects 3 and under had higher volumes than those older than 3. Anterior tracts in subjects 3 and under had an average volume of 0.0084, while anterior tracts in subjects older than 3 had an average of 0.0059. Long tracts did not show a significant interaction with age [*F*_(1, 143)_ = 0.025, *p* = 0.875, η_*p*_^2^ < 0.005]. Subjects 3 and under had an average volume of 0.0059 and subjects older than 3 had an average of 0.0059. Posterior tracts displayed a significant interaction with age [*F*_(1, 147)_ = 78.112, *p* < 0.005, η_*p*_^2^ = 0.347] indicating that the posterior tracts of the AF in subjects 3 and under had higher volumes than those older than 3. Subjects 3 and under had an average volume of 0.0071, while those older than 3 had an average of 0.0042. The two-way interaction between hemisphere and tract [*F*_(2, 437)_ = 3.208, *p* = 0.041, η_*p*_^2^ = 0.014] and simple main effects testing indicated that the volume of anterior tracts was affected by age. Anterior tracts displayed a significant interaction with hemisphere [*F*_(1, 147)_ = 10.797, *p* = 0.001 η_*p*_^2^ = 0.068] indicating that the anterior tracts of the AF in the right hemisphere had higher volumes than those in the left. Anterior tracts in the right hemisphere had an average volume of 0.0071, while anterior tracts in the left had an average of 0.0061. Long tracts did not show a significant interaction with hemisphere [*F*_(1, 143)_ = 0.597, *p* = 0.441, η_*p*_^2^ = 0.004]. Tracts in the right hemisphere had an average volume of 0.0060 and those in the left had an average of 0.0057. Posterior tracts did not show a significant interaction with hemisphere [*F*_(1, 147)_ = 0.016, *p* = 0.900, η_*p*_^2^ < 0.005]. Tracts in the right hemisphere had an average volume of 0.0051, while those in the left had an average of 0.0050. There was no three-way interaction found among age, hemisphere, and tract [*F*_(2, 437)_ = 0.553, *p* = 0.576, η_*p*_^2^ = 0.003].

## Results summary

### ADC and FA

There were age-related differences across anterior, long, and posterior AF tracts in raw ADC and FA data: 3 and under had higher raw ADC in the anterior and long tracts, while over 3 had higher raw ADC in the posterior tract, and 3 and under had lower FA values in all tracts. Subjects over 3 had higher WB-normalized FA values in all tracts.

In all ages, raw and WB-normalized FA values were higher in the long tract than those in the anterior tract, and WB-normalized FA values were higher in the long tract than those in the posterior tract. Also in all ages, raw and WB-normalized ADC values were higher in the posterior tract than those in the anterior and long tracts.

### Length, tract count and volume

There were age-related differences across anterior, long, and posterior AF tracts in raw and in WB-normalized length, tract count, and volume data, except for some measurements in the posterior tract: Subjects older than 3 had higher raw length, tract count, and volume values, as well as higher WB-normalized length in all tracts except for the posterior tract (no age effect). However subjects 3 and under had higher WB-normalized tract count and volume measurements in all tracts except for WB-normalized volume in the long tract (no age effect).

In all ages, raw and WB-normalized length was greater in the long tracts compared to the other two tracts (long>anterior, long>posterior), while raw and WB-normalized tract counts were the highest in the anterior tract, followed by those in the posterior and long tracts (anterior>posterior>long). Raw volume (anterior>posterior, long>posterior) and WB-normalized volume (anterior>long>posterior) also showed age-independent differences across tracts.

### Hemispheric asymmetry

In all tracts, hemispheric asymmetries were found in raw (left<right) and WB-normalized (left>right) tract count and raw volume (left<right). In raw and WB-normalized length, and WB-normalized volume, rightward asymmetry (left<right) was found only in the anterior tract; other tracts were not significantly affected by hemisphere. See scatter plots for hemispheric asymmetry in Supplementary Figure S8.

## Discussion

Using HARDI tractography, we identified anterior, posterior, and long segments of the arcuate fasciculus (AF) in each cerebral hemisphere of 83 healthy subjects ranging from 40 gestational weeks (GW) newborns to 28-year-old adults. The growth and laterality patterns of the three AF segments were assessed by tract count, volume, length, fractional anisotropy (FA), and apparent diffusion coefficient (ADC) values. Comparing subjects 3 and under to older subjects, we found that the three segments of the AF took differential developmental courses. The anterior and long AF tracts showed lower ADC values in subjects over 3 years old as expected, while the posterior tract showed higher ADC in that age range. The posterior tract did not show age-related effect in terms of FA, tract count, length, and volume. These results suggest that the posterior AF tract shows a matured state, indexed by most of the used MRI-based measurements in early postnatal developmental ages, and ADC is a measurement that can detect further maturation of the posterior tract. Interestingly, although tract count normalized by whole brain (WB) tract count showed leftward hemispheric asymmetry in all AF segments, overall rightward hemispheric asymmetry was observed in raw tract count and WB-normalized length and volume. While a number of previous studies have observed a leftward asymmetry in the AF, rightward asymmetry has also been reported in other studies, and, together with the present report, the results in the literature are likely to reflect differences in the methods used. Our tractography approach with no FA threshold may have benefited the results in this study.

### Differential growth patterns of anterior, posterior, and long segments of the arcuate fasciculus

FA and ADC values are often linked to the degree of myelination (e.g., Qiu et al., 2015). Thus, differential patterns of FA and ADC over the three AF tracts potentially show differential degrees of myelination where higher FA and lower ADC indicate greater myelination. According to the raw and WB-normalized data, the long tract often showed higher FA and lower ADC values than those in the other tracts, which indicated that the long tract may be more myelinated than the other tracts across ages.

Anterior tract showed lower FA values than those in the long tract in both the raw and the WB-normalized data. This may explain why other research studies using FA threshold almost always found the long tract but often did not observe the anterior part of the AF. For example, Brauer et al. (2013) reported that frontal regions of the AF were especially difficult to detect by tractography, as the AF had still not yet fully matured, indexed by significantly lower FA even at age 7. Thus, it is critical not to use an FA threshold to detect the AF when studying full developmental trajectories in newborns (see also below “*Tractography approach with no FA threshold*”).

While the anterior and long AF tracts showed expected lower ADC values in subjects over 3 years old, the posterior tract showed higher ADC in that age range. Regarding FA, tract count, length, and volume, the posterior AF tract did not show age-related effect. These together suggest that the posterior AF tract shows a matured state indexed by most of the used MRI-based measurements in early postnatal developmental ages, and ADC is a measurement that can detect further maturation of the posterior tract. Higher ADC was observed in the posterior AF in subjects over 3 years old compared to under 3 years old, but it was only found in the raw AF data and not in the WB-normalized data. The reason for this would be that changes of ADC values in the posterior AF are parallel to the overall changes of the brain development. However, it is well established that ADC values in general decrease during development, mainly because of myelination. Given that the other AF tracts showed different changes before and after 3 years old, it is possible that other factors also affected the interesting raw and WB-normalization data of the ADC values in the posterior AF. Nevertheless, unlike tract length or volume, FA and ADC are not obviously affected by the brain size. Therefore, the increase of raw ADC values of the posterior AF after 3 years old already has an interpretable meaning as discussed above.

### Tractography approach with no FA threshold

Catani et al. (2007) studied the AF in adults (*n* = 40). Their data points for the long segment are fewer (*n* = 28) than those for the anterior and posterior segments (*n* = 40) (Supplementary Figure S4 in the paper, see below), suggesting they did not find the long segment in all subjects. Our current study successfully identified the long pathways in all the adult subjects studied, as well as in younger subjects except for those with motion artifacts (7 out of 90 subjects: four newborns/infants under a year old, one 3 year old, one 6 year old, and one 9 year old).

**Figure 4:**
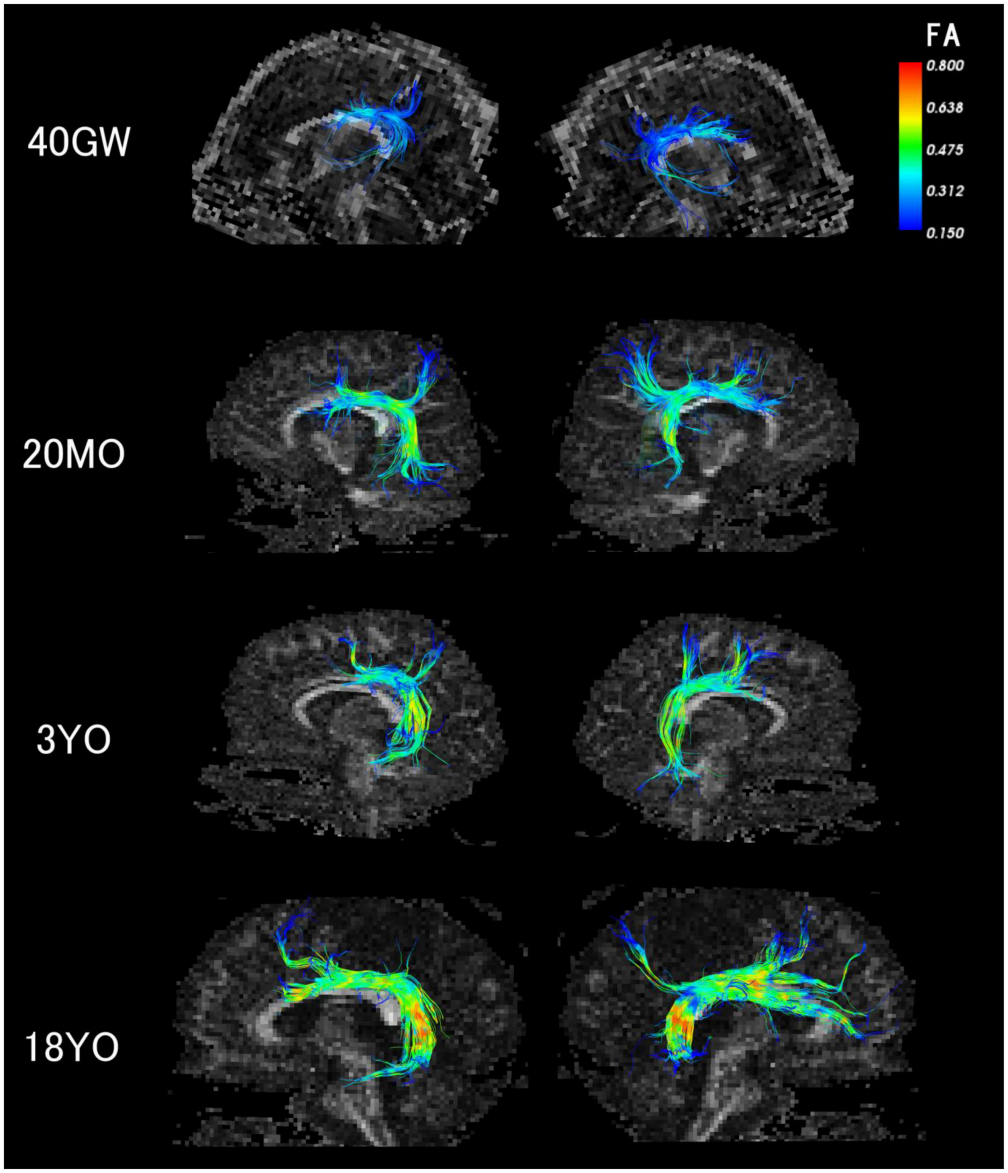
Color-corded FA values on detected AF pathways in 40 gestational weeks, 20 months old, 3 years old, and 18 years old subjects. The color coding of fibers is based on FA values.

It is still possible that there are neurologically healthy people with very small or no AF in the right hemisphere; it is just that our current subject population was made up of people who had the AF in the both hemispheres. In the context of literature, the difference in methods causing the differences in results is another possibility that explains our results. Major points of differences between our two studies in DTI methods includes their use of a 1.5T scanner, an FA threshold (0.2), and spatial resolution 2.5mm isotropic. Although the majority of the AF long segment has FA values of over 0.2, there are certain regions where FA is certainly below 0.2, or close to/just above 0.2. In some subjects, the relatively low FA regions are located throughout the long segment, especially where it branches (Figure 4). Therefore it is possible that the long segment was not identified consistently in their study.

### Hemispheric asymmetry of the arcuate fasciculus

Although the literature often showed leftward hemispheric asymmetry in the AF (Büchel et al., 2004; Nucifora et al., 2005; Parker et al., 2005; Powell et al., 2006; Rodrigo et al., 2007; Vernooij et al., 2007), there are other studies that reported rightward asymmetry of the AF (Barrick et al., 2007; Catani et al., 2007; Thiebaut de Schotten et al., 2011a,b; Häberling et al., 2013; Badisavljevic et al., 2015). Several research groups have measured tract count by segmenting the AF into posterior, anterior, and long tracts in a similar manner to the current study (but with FA threshold) in adults (18–22 years old) (Thiebaut de Schotten et al., 2011b) and subjects aged 9–40 years old (Budisavljevic et al., 2015). They found that tract count of the anterior tract (rightward) and the long tract (leftward) showed hemispheric asymmetry by 9 years old and through adulthood, while the posterior tract showed rightward asymmetry by/around 9 years old and became bilaterally symmetrical during adolescence. In the current study, in terms of tract count, raw data showed overall rightward, and WB-normalized data showed overall leftward hemispheric asymmetries with no age effect. This difference can be due to several reasons, but one possibility is the difference in methods used in our and their studies (i.e., FA threshold, for example, see the previous section). Another possibility is the difference in age distributions studied, and it may be necessary to assess continuous age effect on the AF development, instead of grouping by age range as we did in this study.

Our results showed overall rightward asymmetry in raw volume data, while only the anterior tract represented rightward asymmetry in WB-normalized volume data. This result suggests that the AF volume overall is larger in the right hemisphere, but compared to the whole brain development, the AF sub-tracts show differential laterality patterns: the long and posterior tracts in the both hemispheres develop proportionally to the whole brain, while the right anterior tract acquires greater volume compared to the left anterior tract, taking into account the whole brain development.

Whether a study uses FA threshold or not also likely affect the results including hemispheric asymmetries. For example, Dubois et al. (2009) found a leftward asymmetry of the AF in newborns using DTI tractography. Important differences between their study and our present and previous (Song et al., 2014) studies are not only that they used DTI while we used HARDI, but also that they used an FA threshold (< 0.15) for tracking pathways. As we explained in the method section, we do not use an FA threshold in our studies in order to identify immature pathways. It is possible that, by using such a threshold, early-matured parts of the AF with higher FA values showed leftward asymmetry in their study, but together with other, more immature parts of the AF, their findings could be different. Dubois et al. (2009) actually recognize that they had difficulty finding pathways in the anterior parts of the fasciculus, and their identification of the AF looks very similar to a posterior part of the long segment in our current study. Dubois et al. (2009) recruited 23 subjects ranging from 3.9 to 18.4 postnatal weeks, while we included 17 subjects from birth (ranging from 1 postnatal day to a year old) and 7 subjects between the ages of 1 and 3.

Past studies have also found that there is leftward asymmetry in the planum temporale, with this asymmetry being correlated with reading abilities (Foundas et al., 1994, 2004; Nakada et al., 2001; Dehaene-Lambertz et al., 2006; Bloom et al., 2013). Given the evidence that some leftward asymmetries of the AF found with an FA threshold are correlated to language skills, it is debatable whether our method of not using an FA threshold is superior to the other methods in terms of the ability to identify biomarkers of behavior linked to the AF. Future studies are necessary to further assess tractography methods with no FA threshold; it is possible that different types/subtypes of behavior are correlated to biomarkers in regions with low FA values.

### Limitations of the current study

The literature provides many examples of significant developmental changes, especially in regards to social and cognitive aspects, which occur around 3 years of age, during the toddler stage (Howes, 1983; Eckerman and Didow, 1989; Willard, 1989). Erikson's psychosocial stages of development may correlate with the functional and structural changes occurring in the brain during major developmental stages. The initiative vs. guilt stage begins at age 3 and describes the child as becoming more assertive and more engaging with peers. According to Piaget's stages of cognitive development, at 3 years of age, a child's mind begins to integrate the use of symbols as language skills become more complex and as memory skills and imaginative thinking increase. The Centers for Disease Control and Prevention provide checklists of major developmental milestones for a 3 year old, which includes increased language and communication skills and more advanced cognitive abilities (American Academy of Pediatrics, 2009).

These major milestones influenced our decision to split our subjects into two groups, one group being 3 years and younger and the other being any subjects older than 3 years old, to take into account the development changes seen in 3 year olds. Although previous tractography studies comparing infant and adult pathways have used similar methods of analysis in order to compare the two sets of values (e.g., Dubois et al. (2008) performed comparative analyses of the maturation state, speed, and stage and Kulikova et al. (2015) used calculations of the Mahalanobis distance in their study of white matter maturation), there may be other growth patterns that occur throughout the lifespan or at different stages of development, other than before and after 3 years old. Taking into consideration the evidence that identified continuous maturation of the human brain through puberty (Chavarria et al., 2014) and past 20 years of age (Li et al., 2014), future studies should look at the more complex growth patterns of the AF pathways in other age groups with gender controll while potentially using additional diffusion properties.

There could be some interactions between diffusion measurements looked at in this study. For example, the length of the pathway could affect the tract count as the number of voxels increases with the length of the pathway. This would lead to a higher calculation of the amount of pathways initiated from each voxel, although this factor can be canceled in our WB-normalization analyses. The results of this study showed that the significant growth in length and tract count were not always coincident, which is a supportive evidence that the effect of the interactions between these measurements are less significant.

The literature regarding handedness and cerebral asymmetry remains conflicting (Geschwind and Levitsky, 1968; Toga and Thompson, 2003; Van der Haegen et al., 2013). Some studies found that handedness played a role in cerebral asymmetry (Snyder et al., 1995; Büchel et al., 2004) while others found that asymmetry did not relate to handedness (Good et al., 2001; Watkins et al., 2001). Whether hand dominance influences white matter tract asymmetry at these early developmental stages also remains unclear. Future studies will aim to take a systematic approach to investigating the development of asymmetry by age, while taking into account other factors, such as gender, language acquisition, and hand dominance.

## Author contributions

MW and ET designed the study. MW, AL, AC, and ET processed and analyzed the data. MW, AL, AG, and ET wrote the manuscript.

### Conflict of interest statement

The authors declare that the research was conducted in the absence of any commercial or financial relationships that could be construed as a potential conflict of interest.
