# Supplementary material for: Detection and Growth Pattern of Arcuate Fasciculus from Newborn to Adult
Source: Front Neurosci. 2017 Jul 14;11:389. doi: 10.3389/fnins.2017.00389 (PMC5509799; doi:10.3389/fnins.2017.00389)
Supplement: Supplementary file 4 [file Image4.PDF]

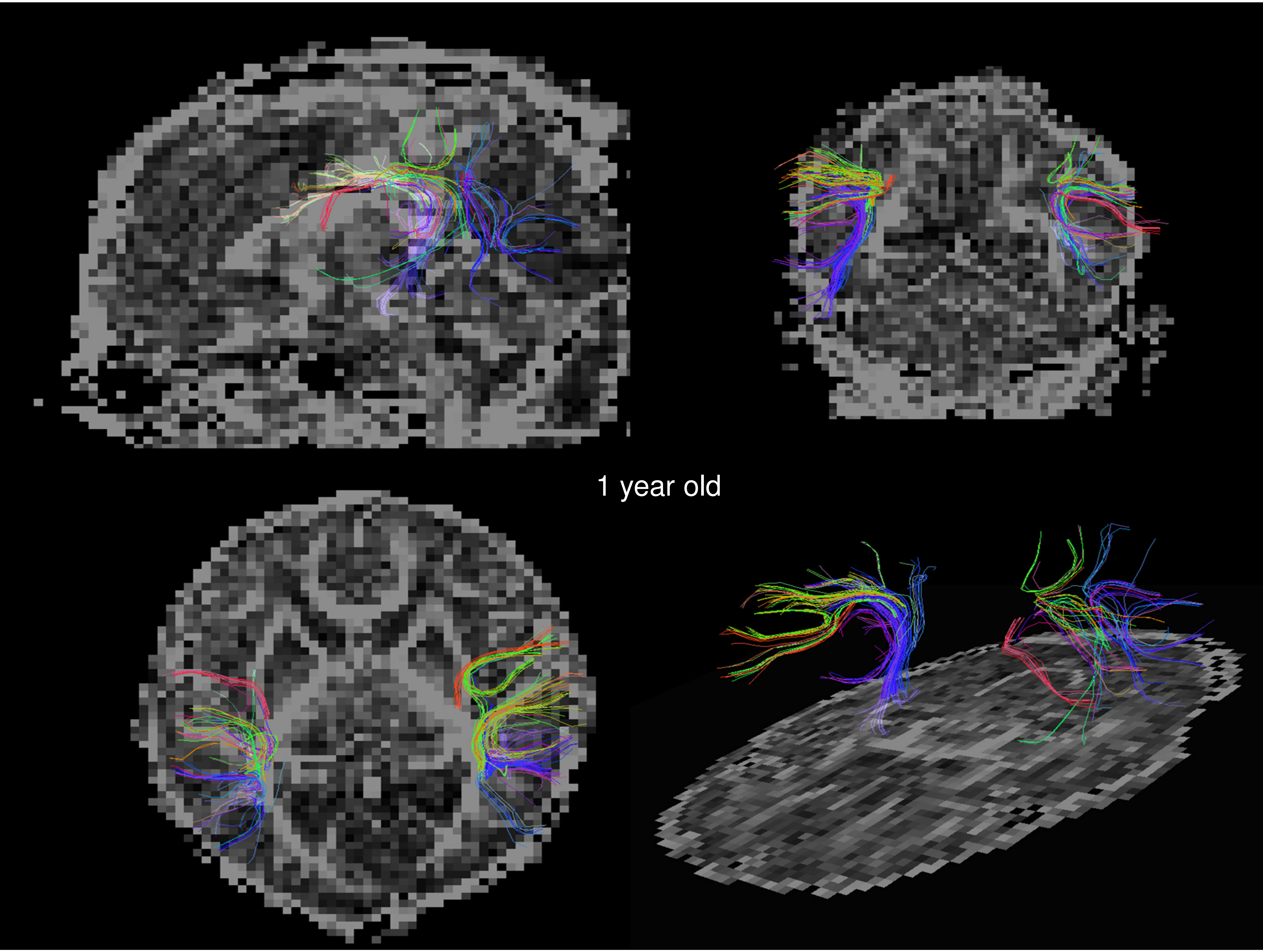
1 year old

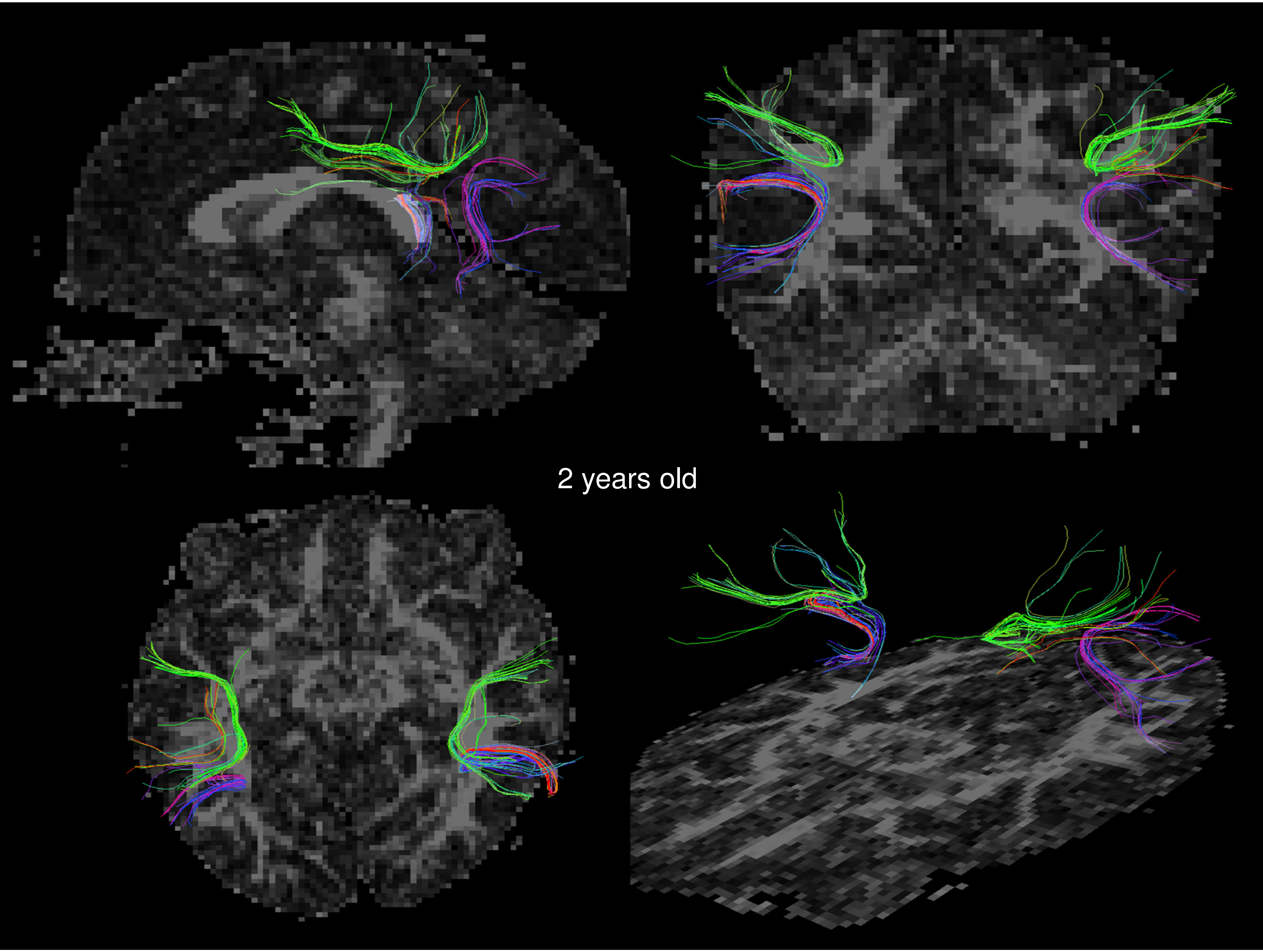
2 years old

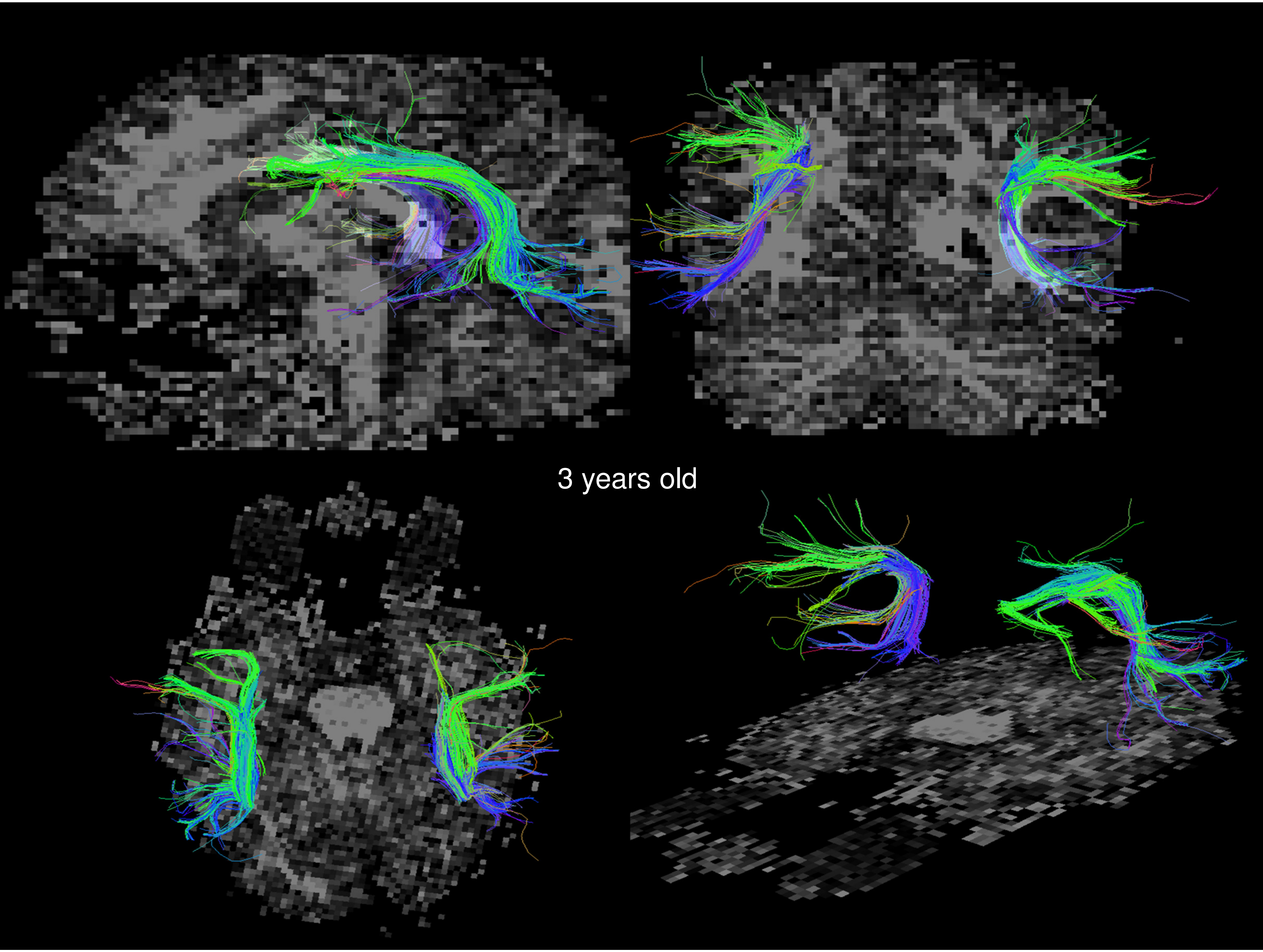
3 years old

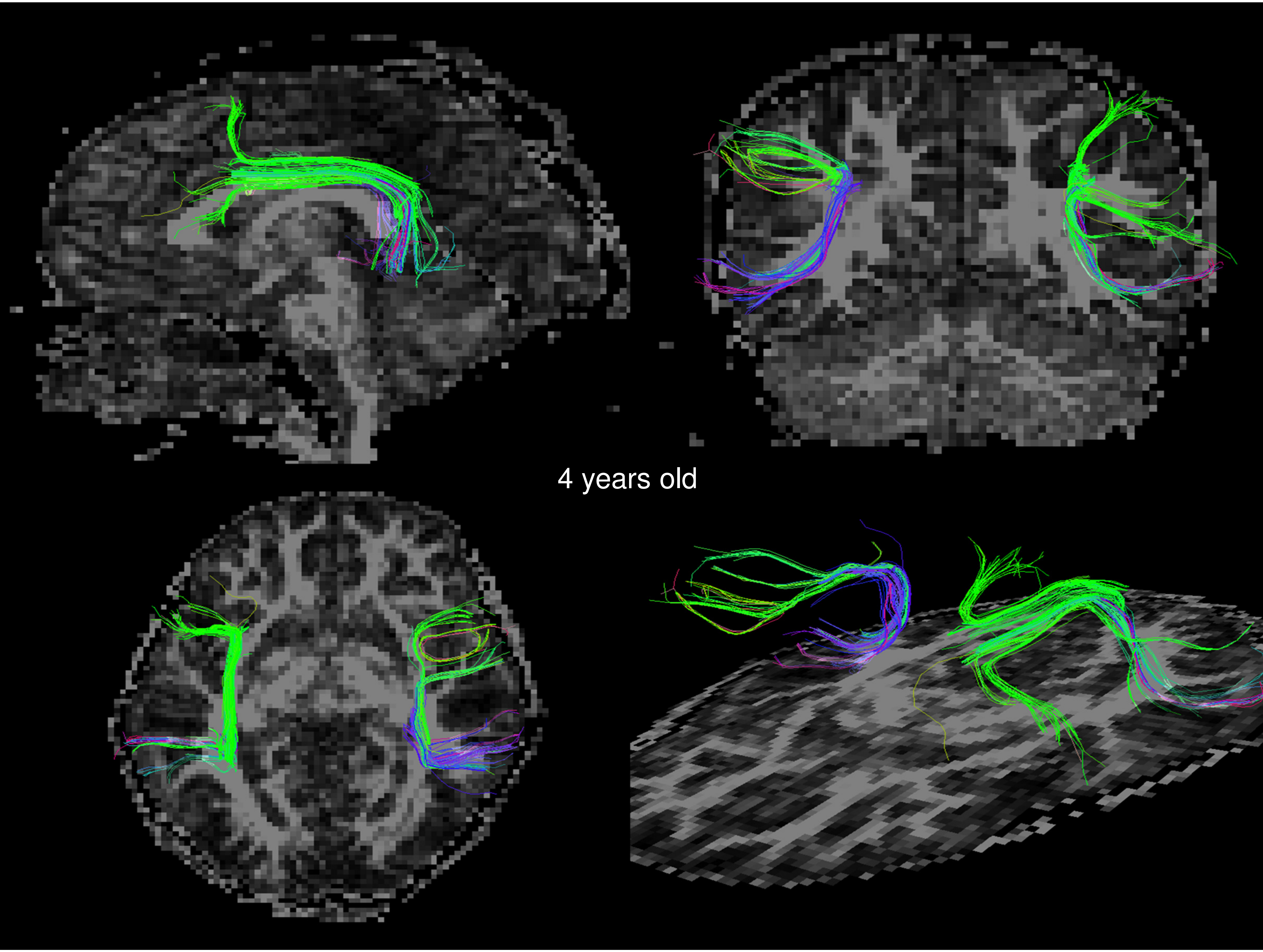
4 years old

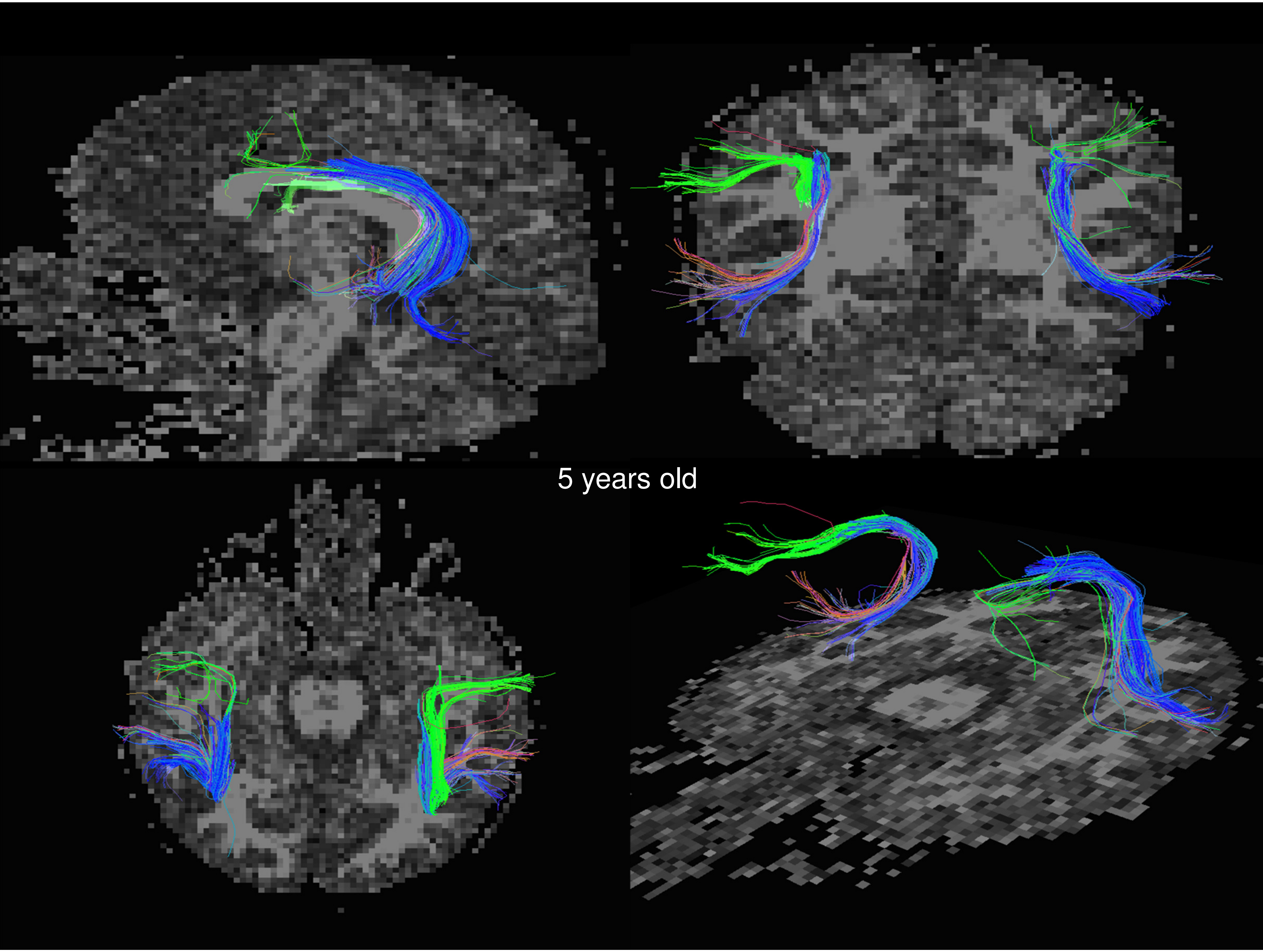
5 years old

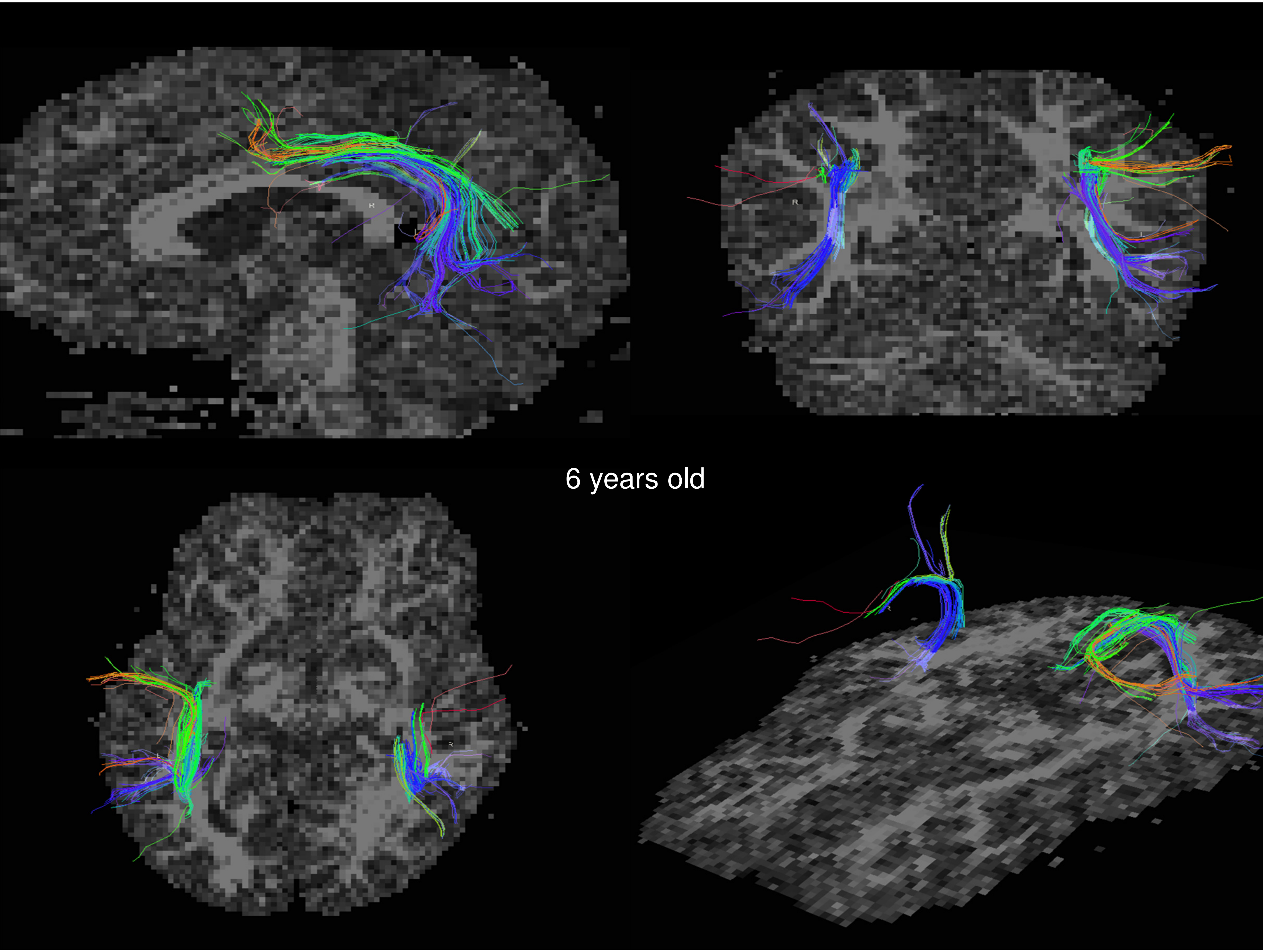
6 years old

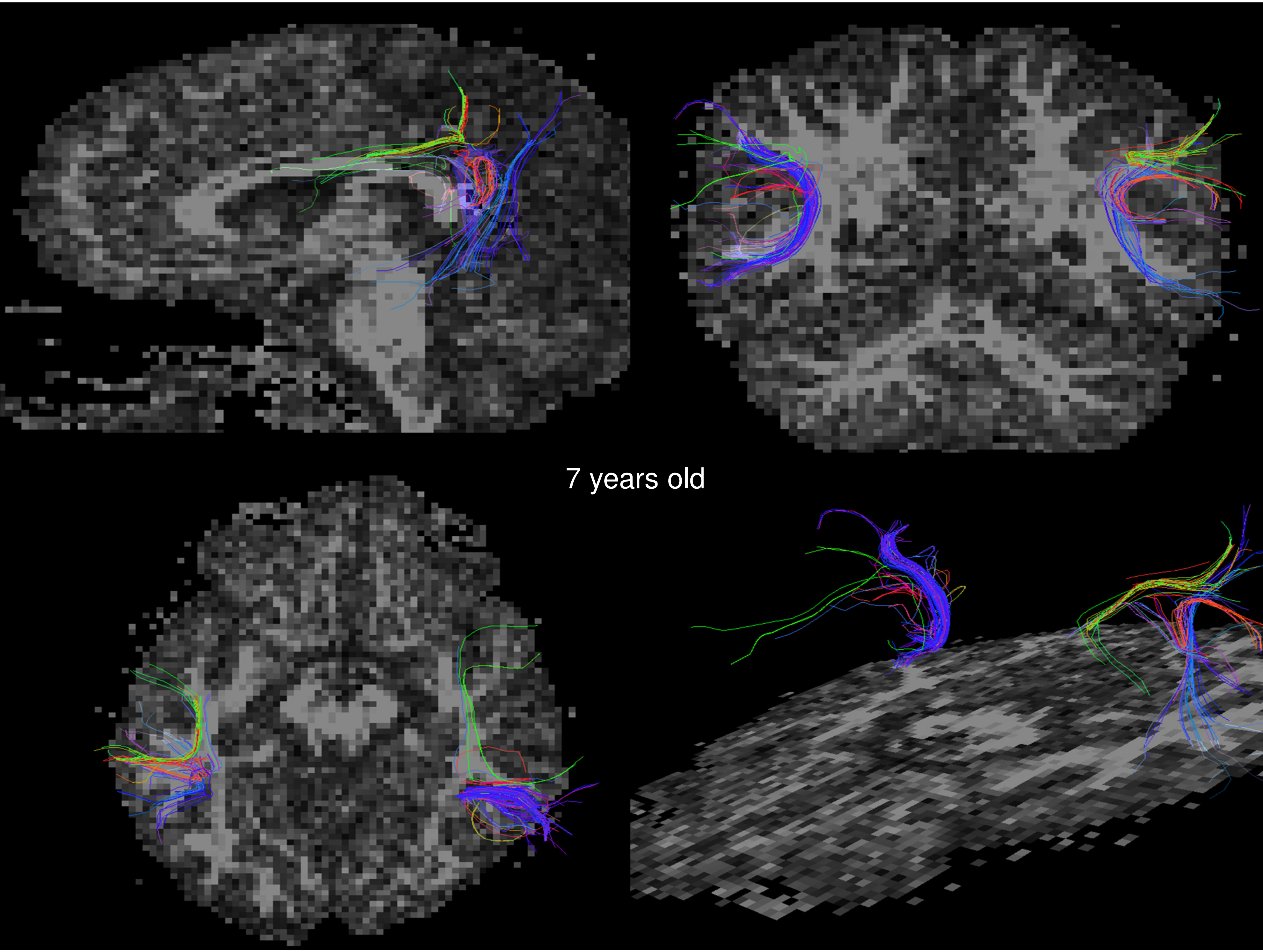
7 years old

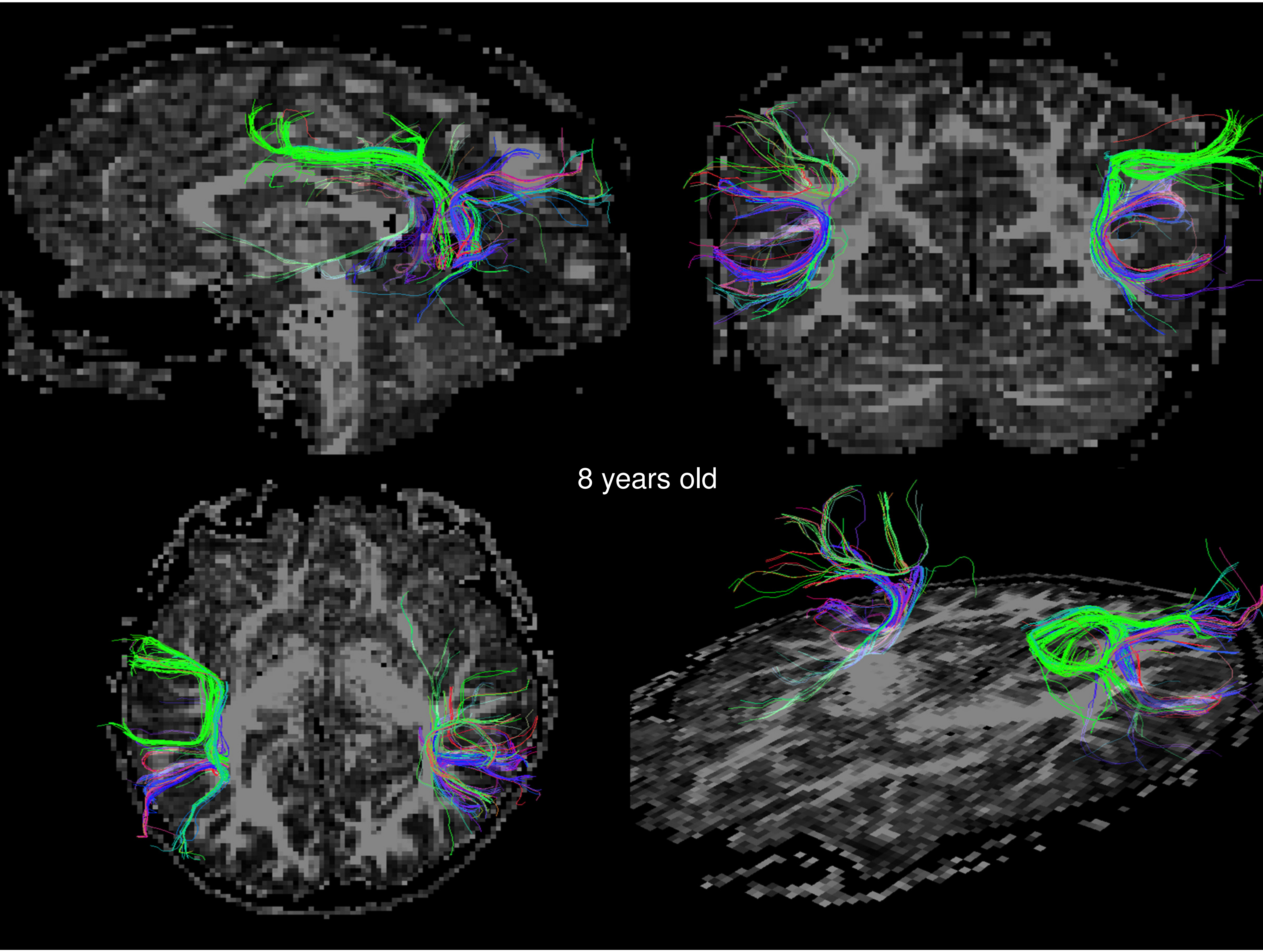

8 years old

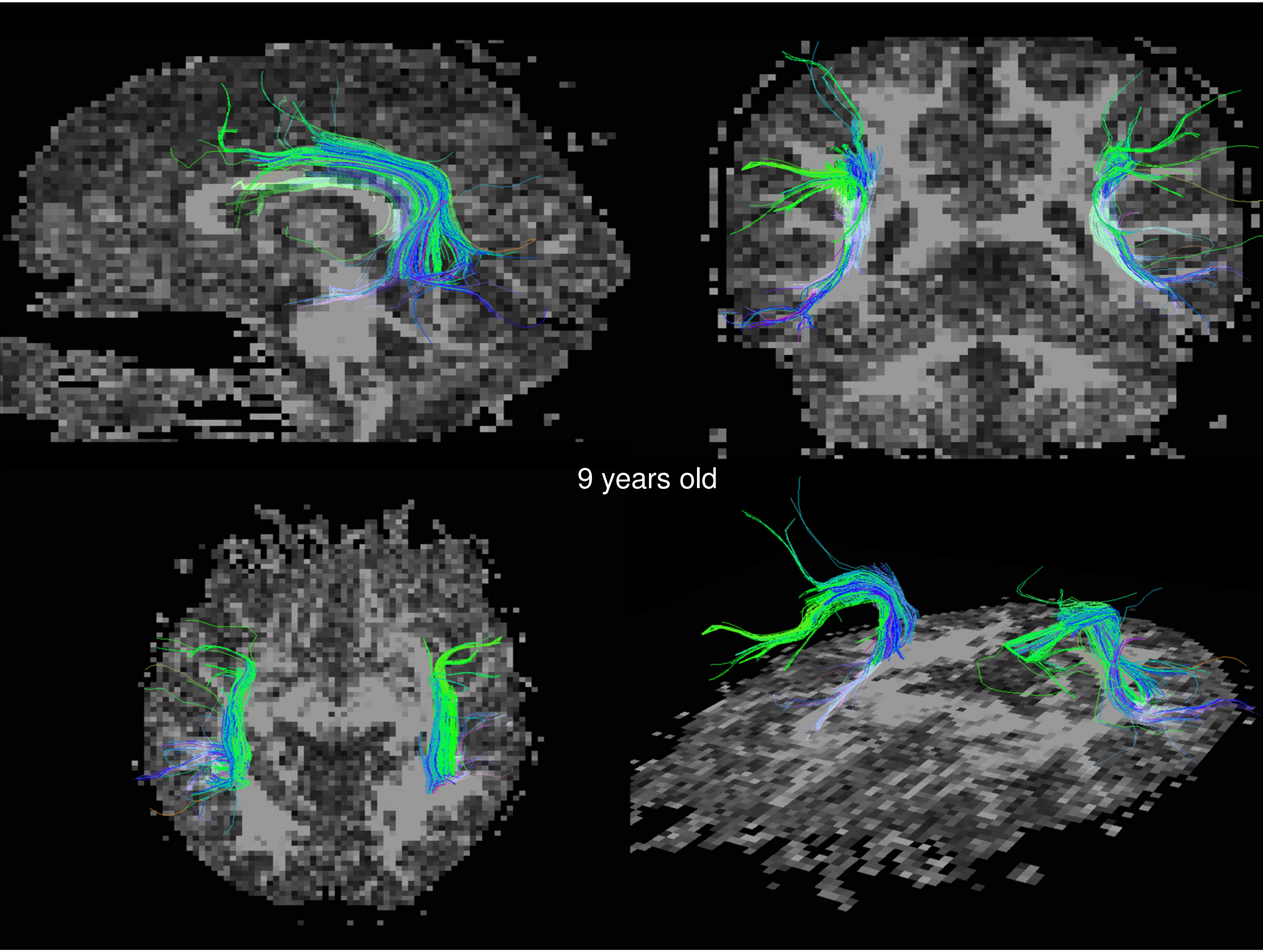
9 years old

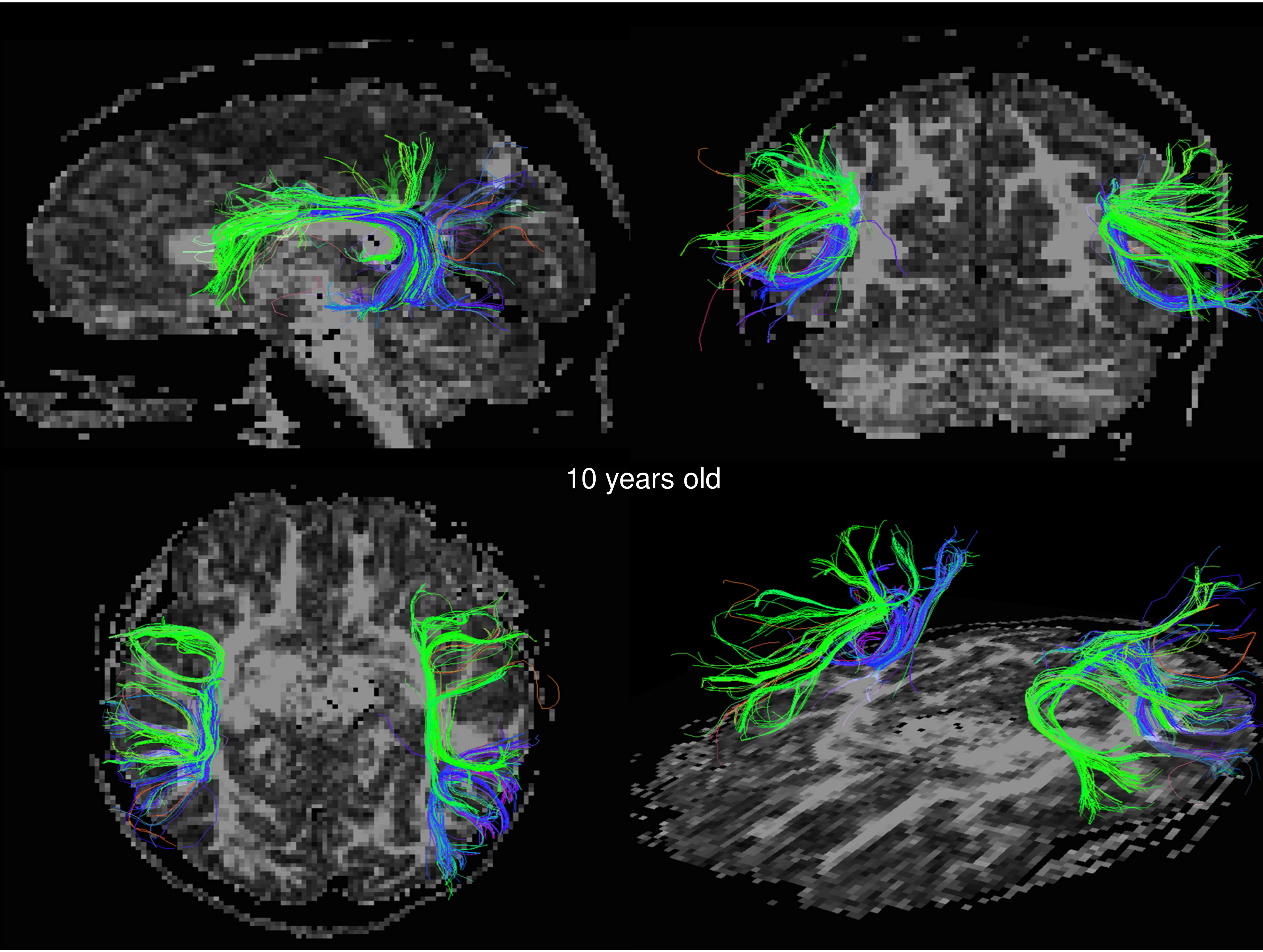
10 years old

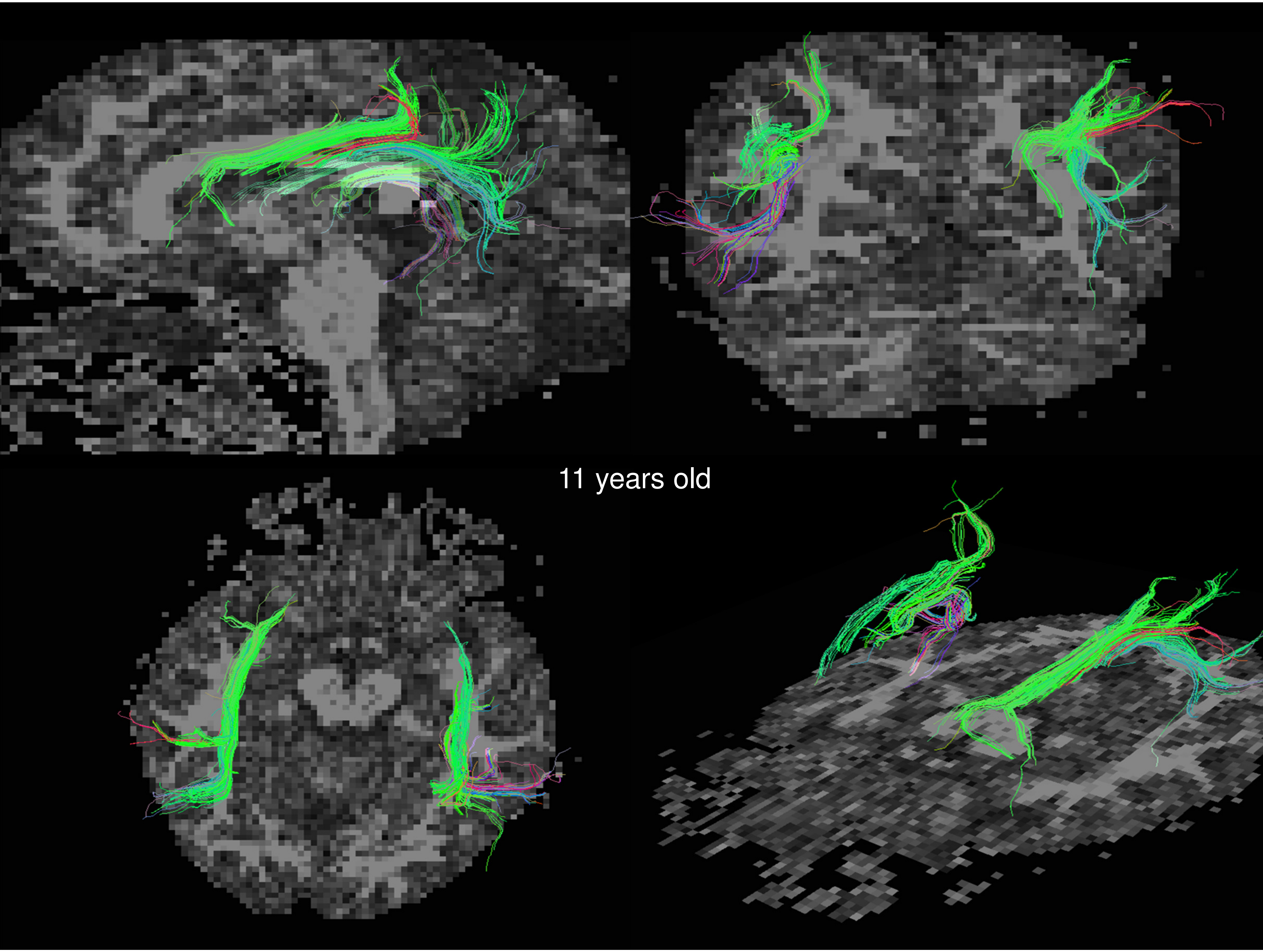
11 years old

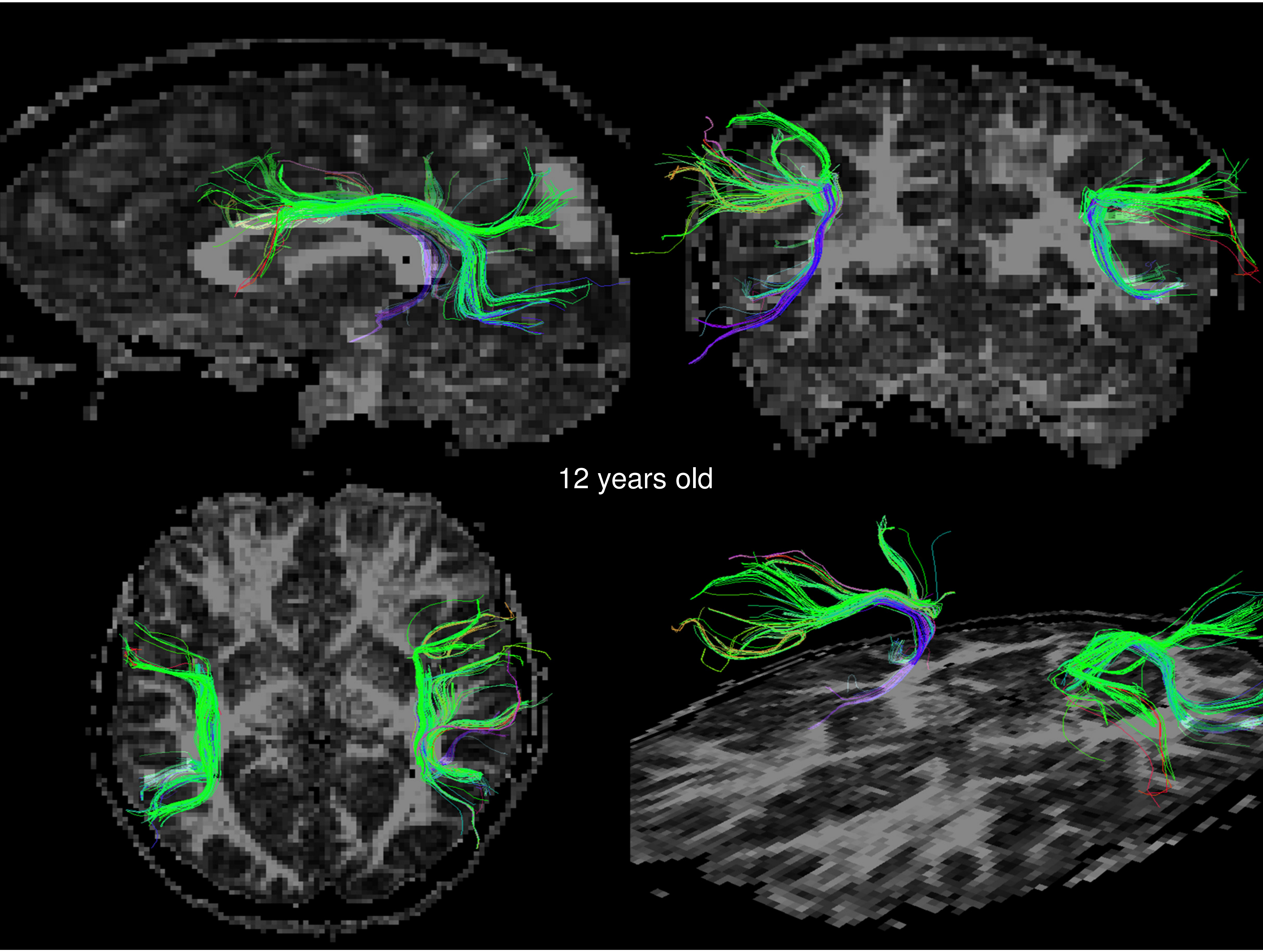
12 years old

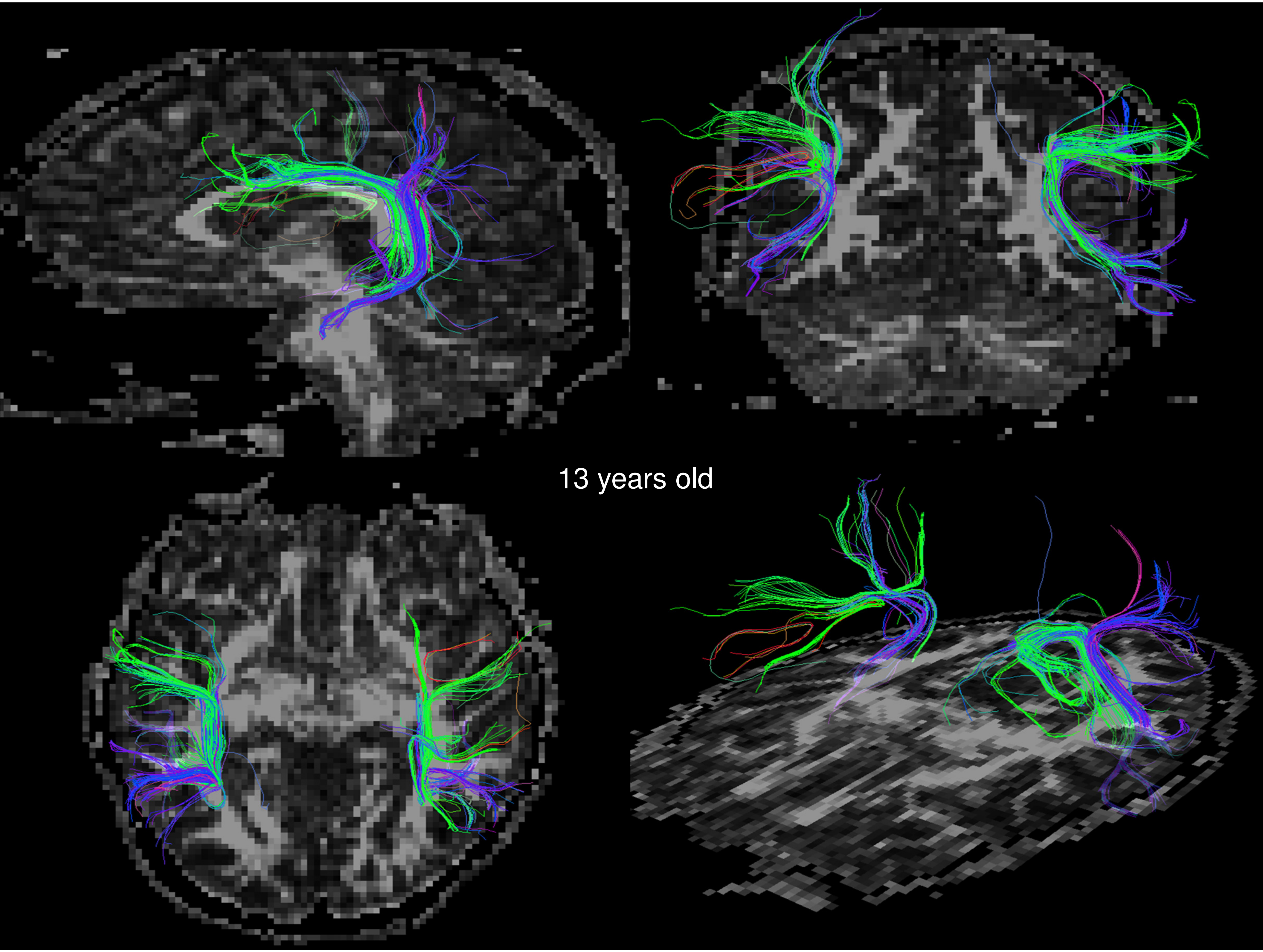
13 years old

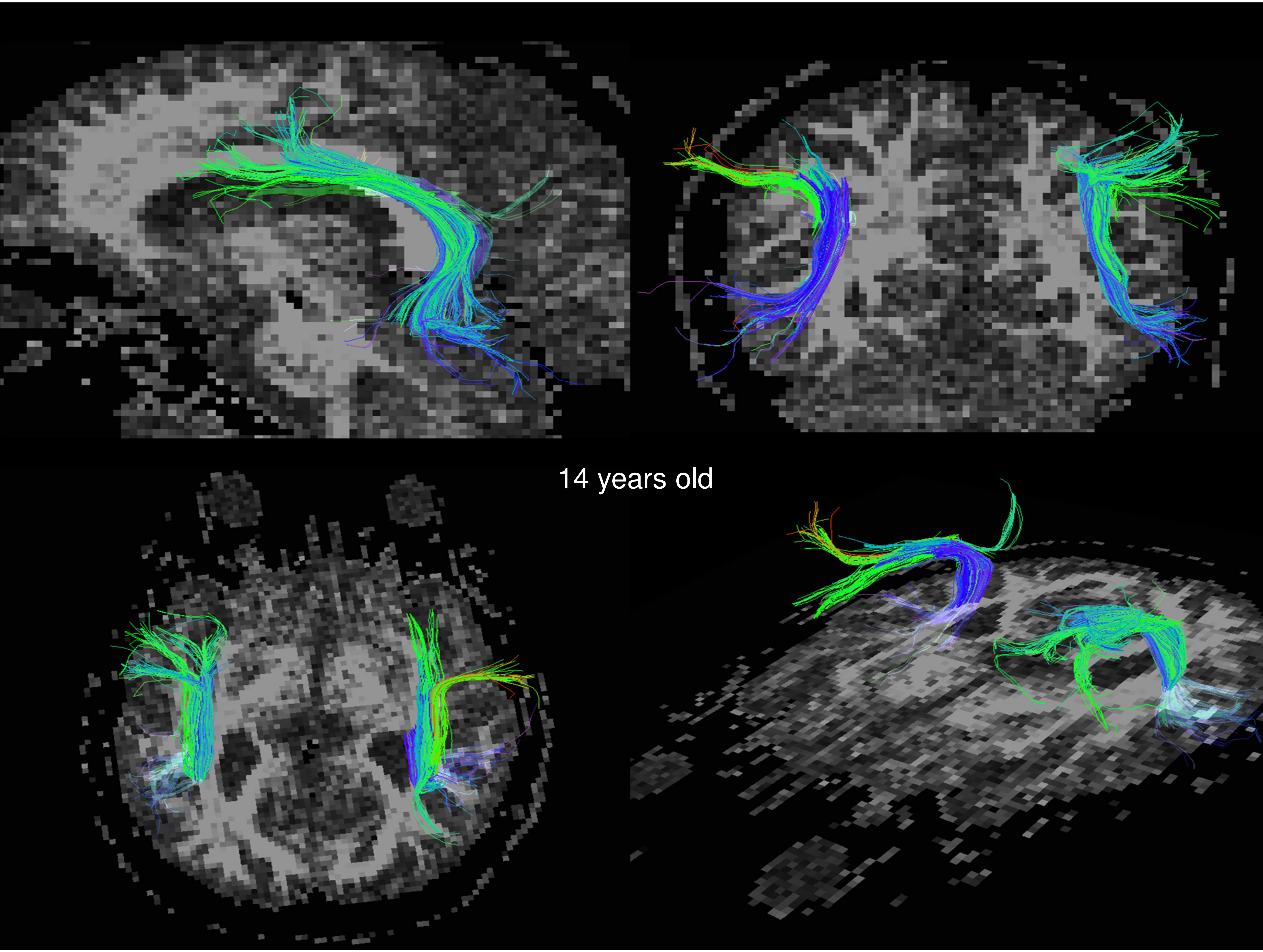
14 years old

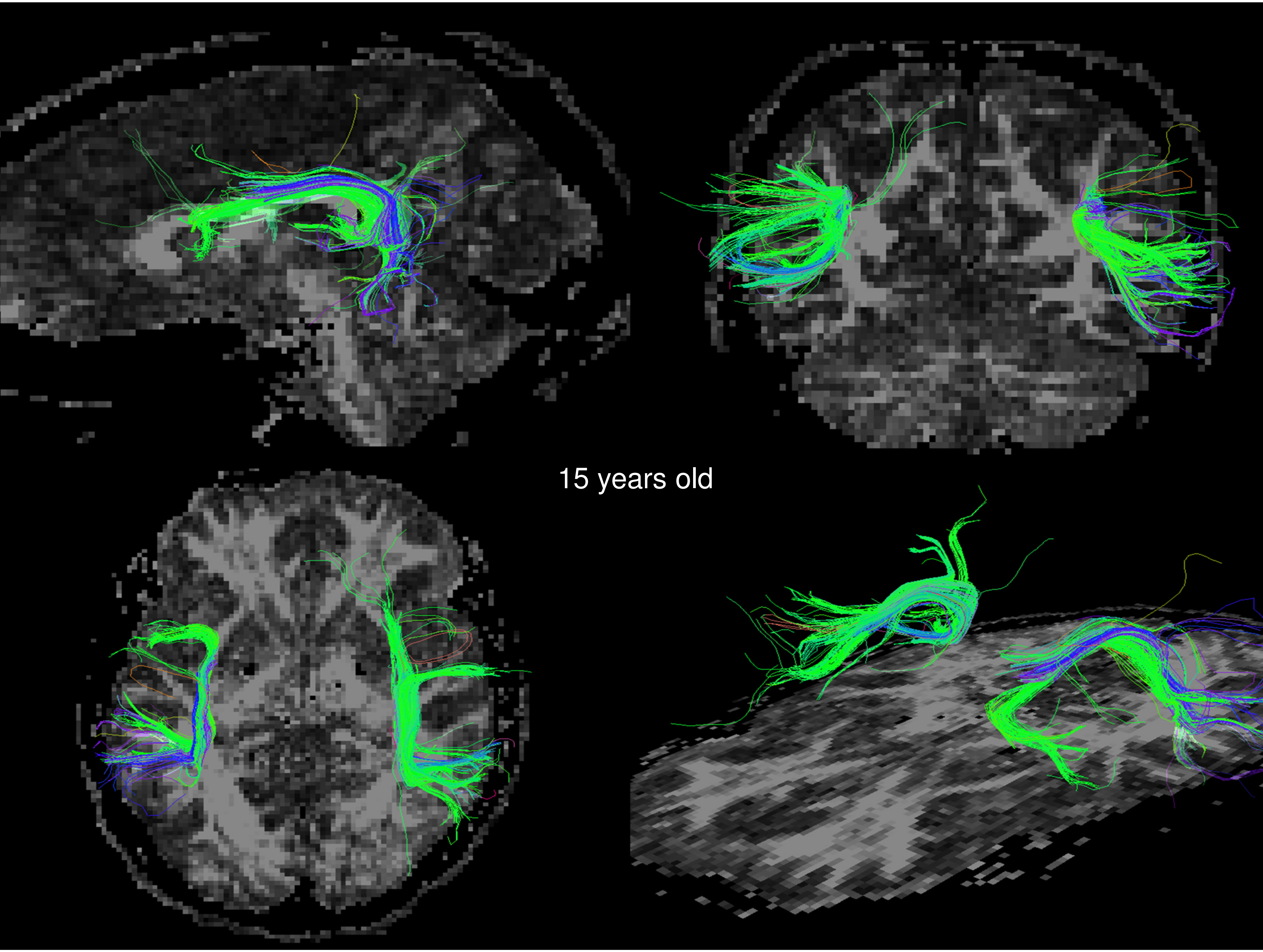

15 years old

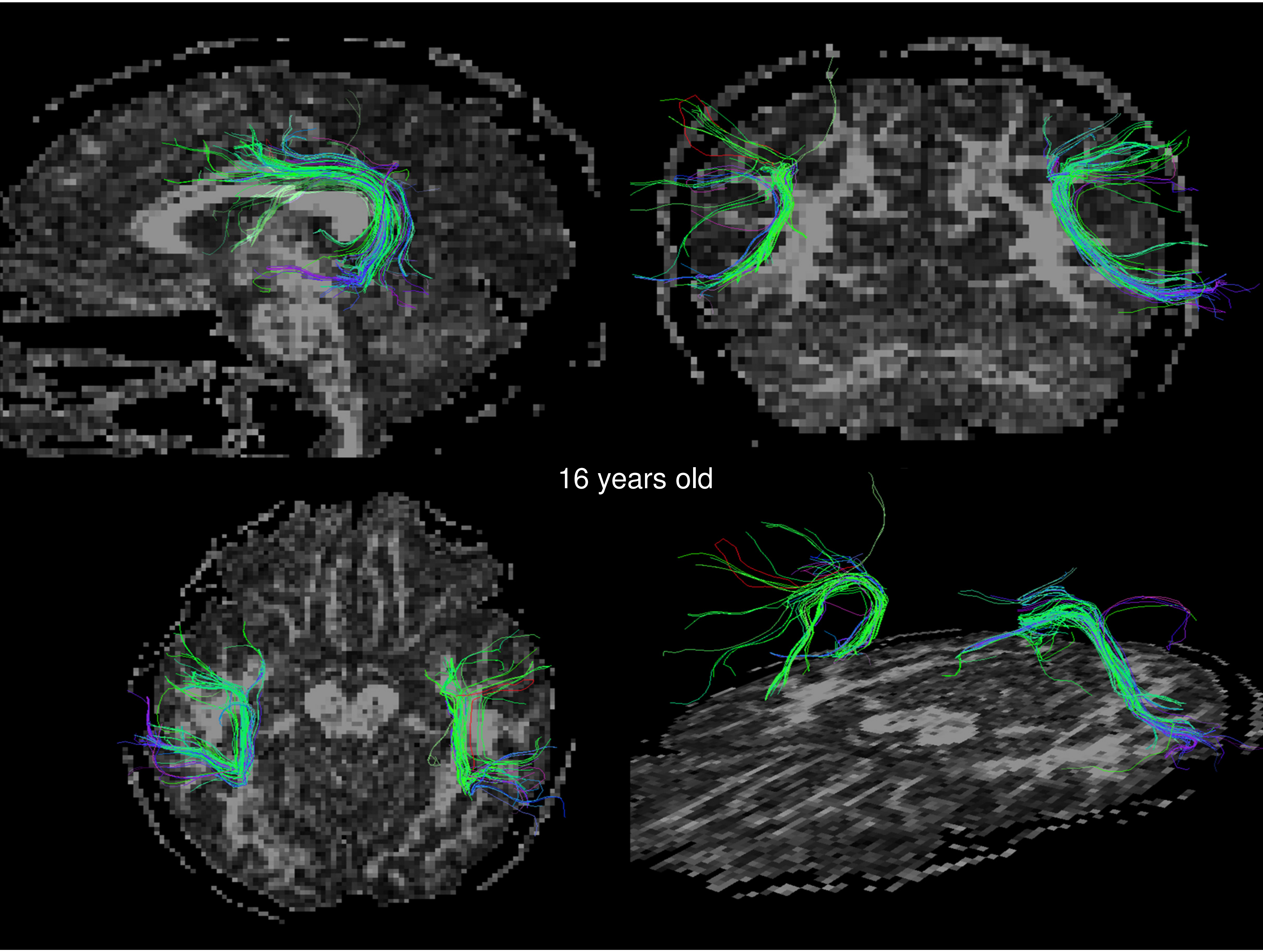
16 years old

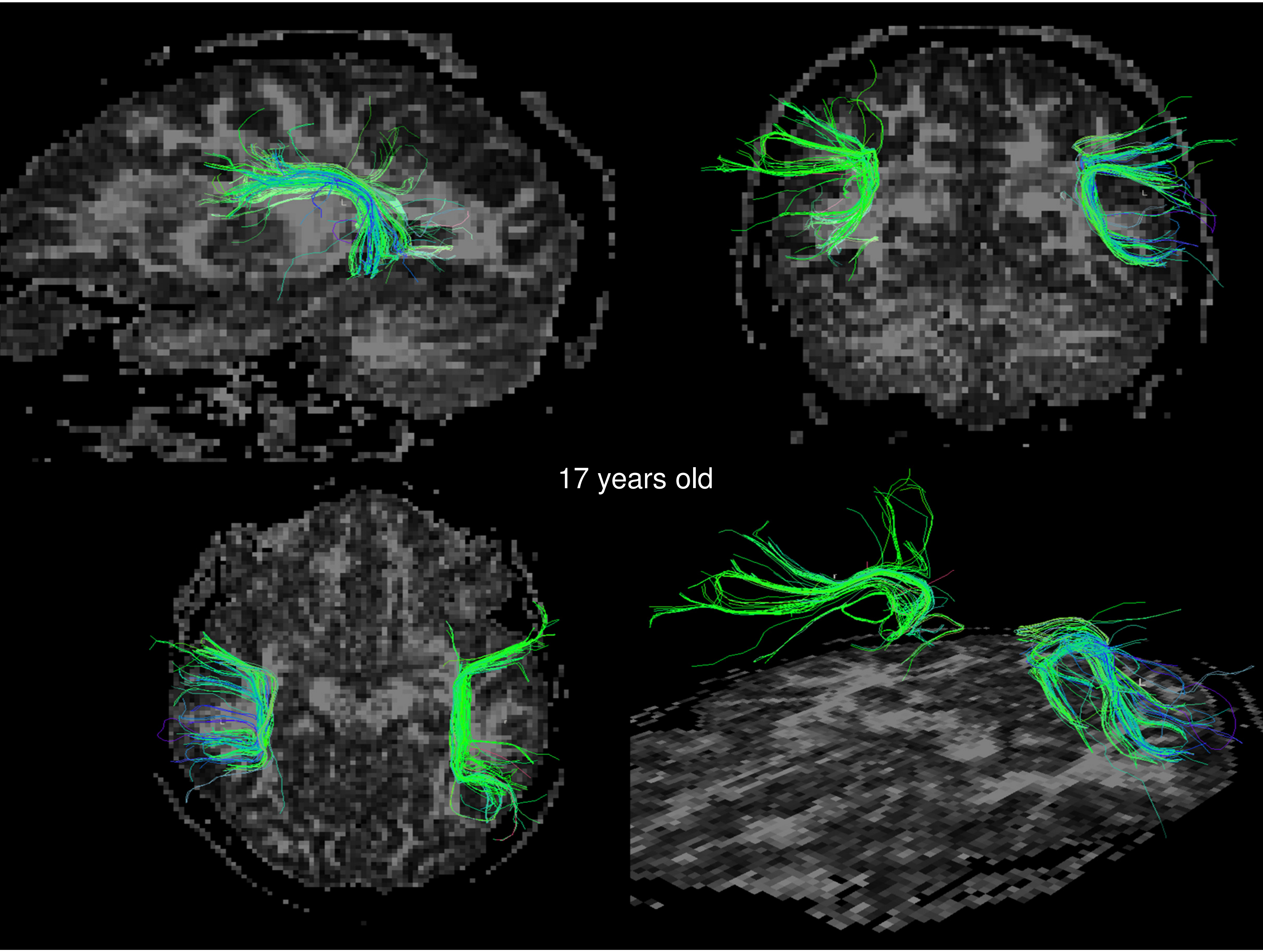
17 years old

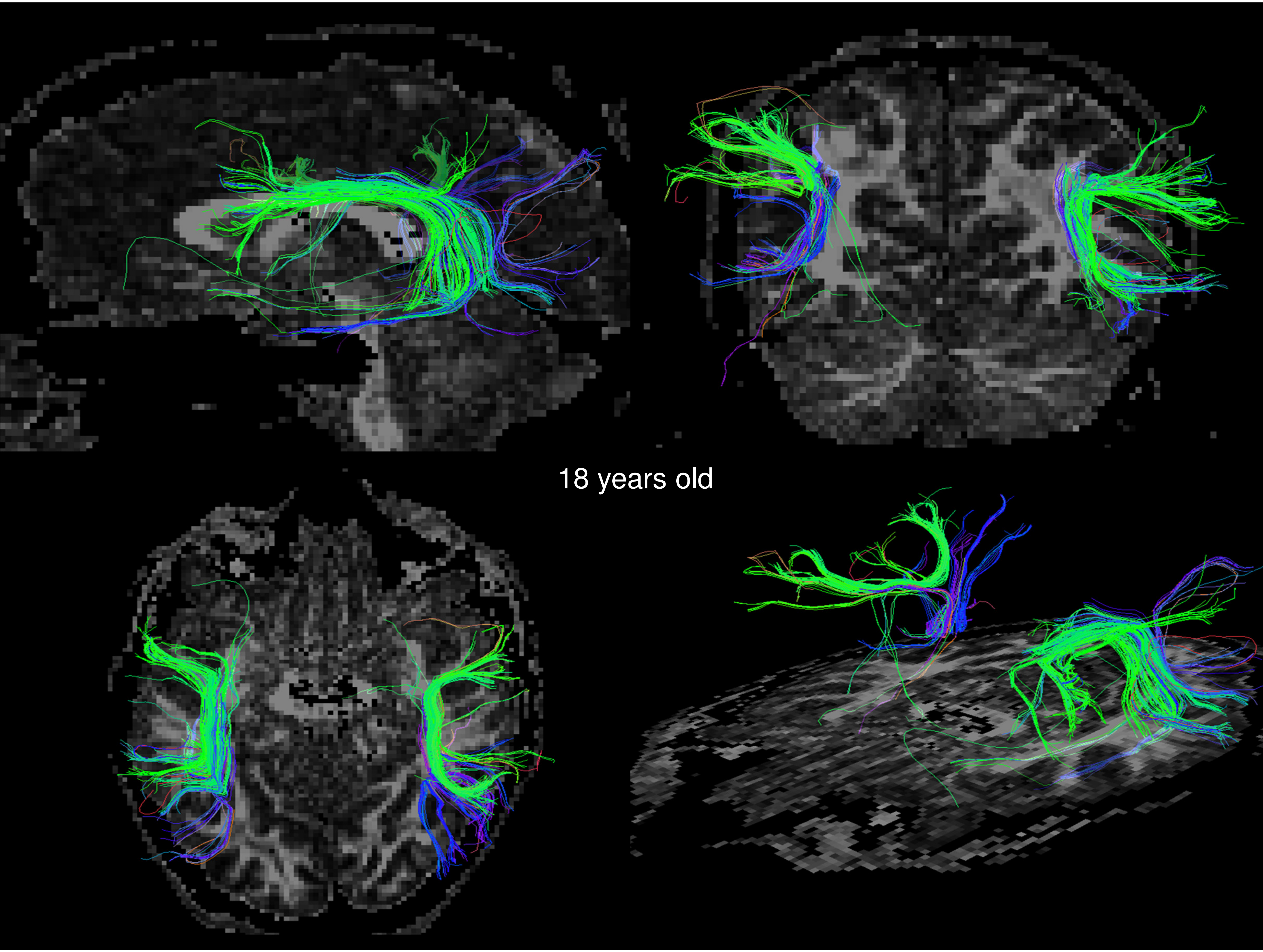
18 years old

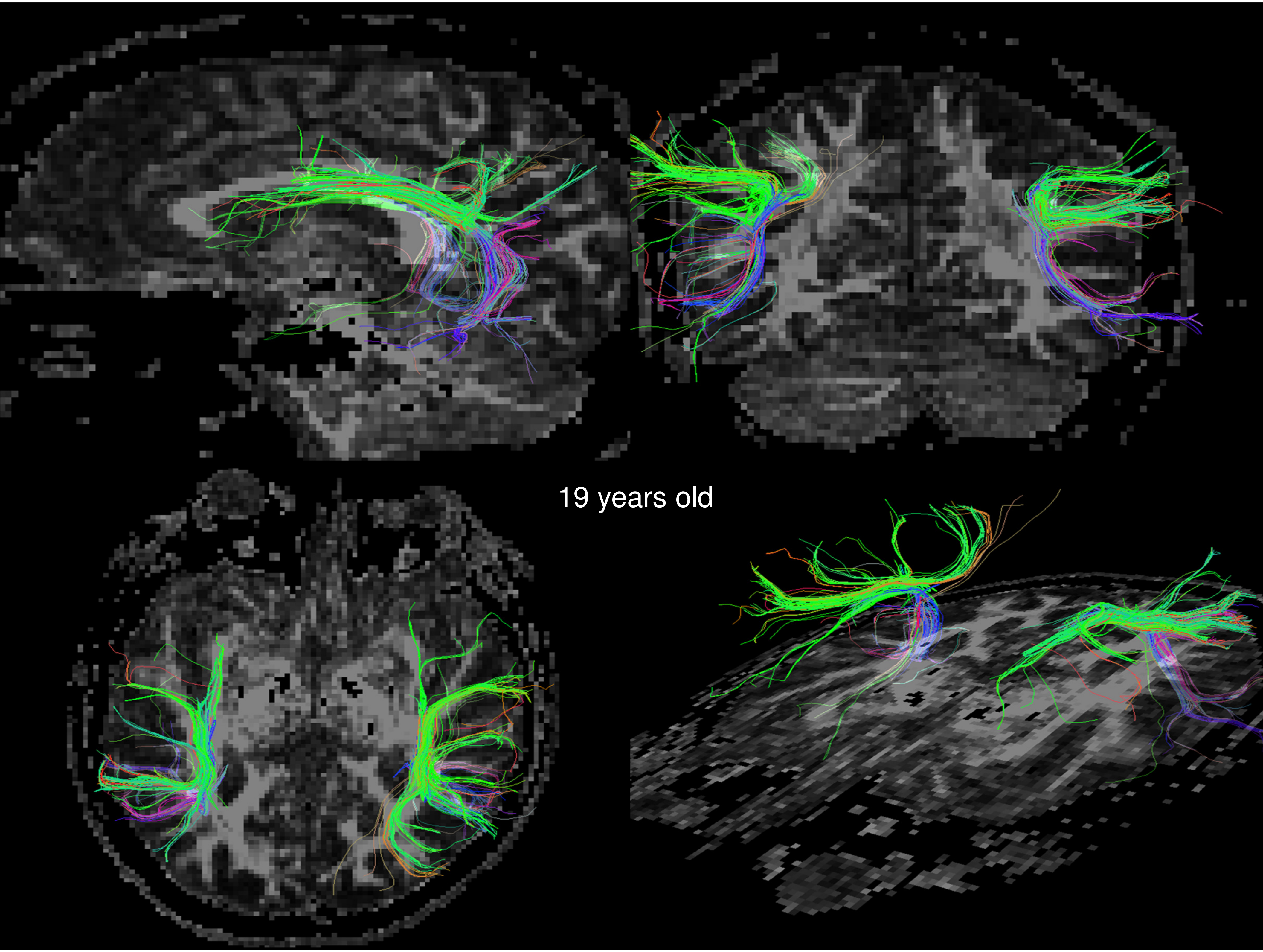
19 years old

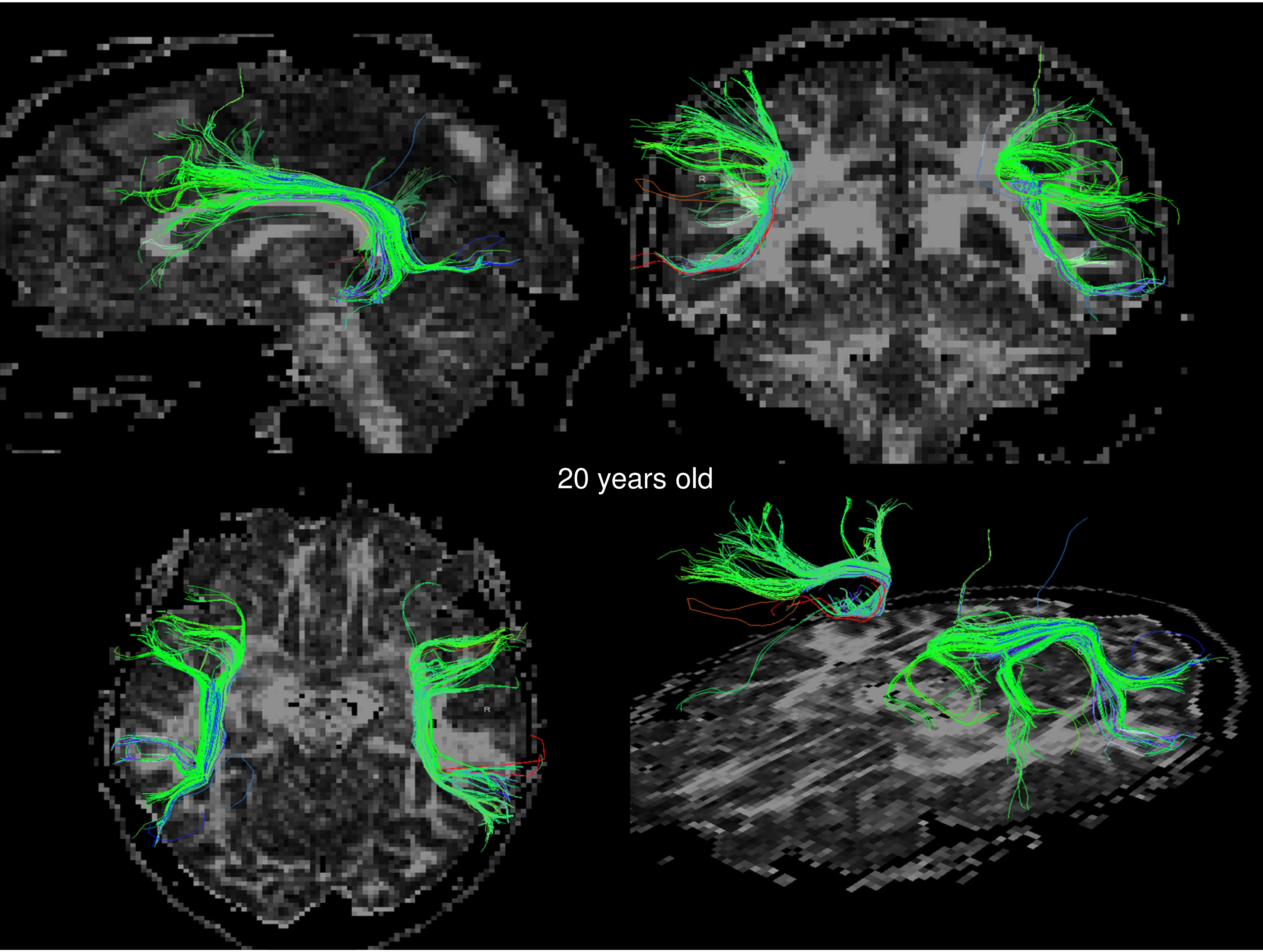
20 years old
