# Supplementary material for: Detection and Growth Pattern of Arcuate Fasciculus from Newborn to Adult
Source: Front Neurosci. 2017 Jul 14;11:389. doi: 10.3389/fnins.2017.00389 (PMC5509799; doi:10.3389/fnins.2017.00389)
Supplement: Supplementary file 5 [file Image5.PDF]

Anterior Arcuate - Raw Data

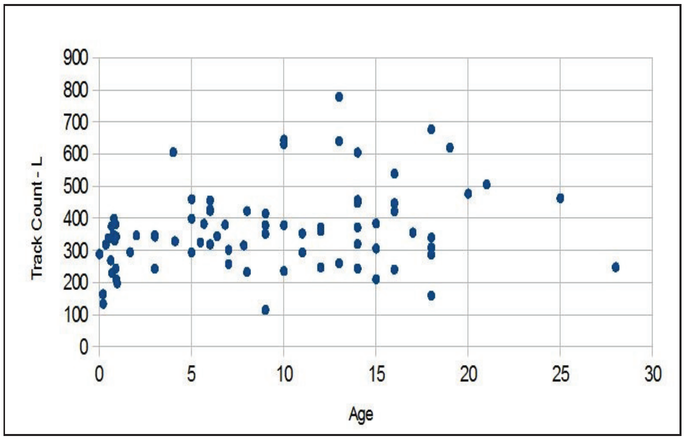

Left Track Count

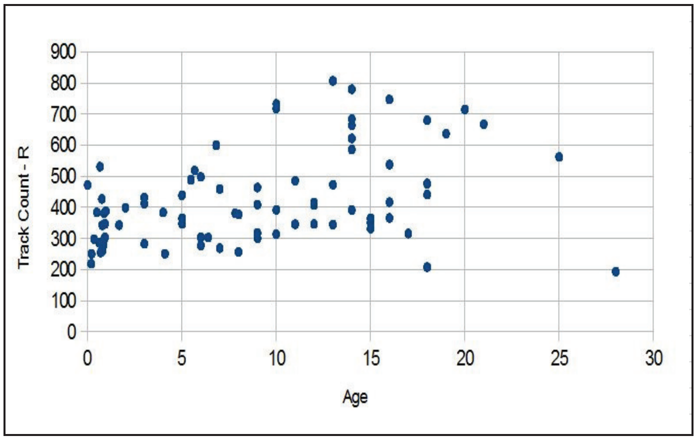

Right Track Count

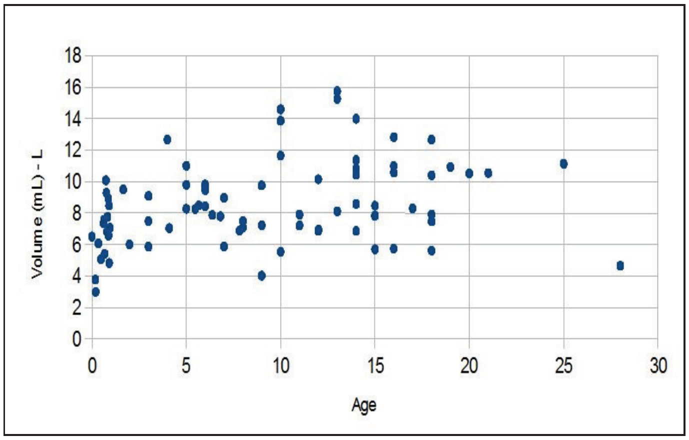

Left Volume

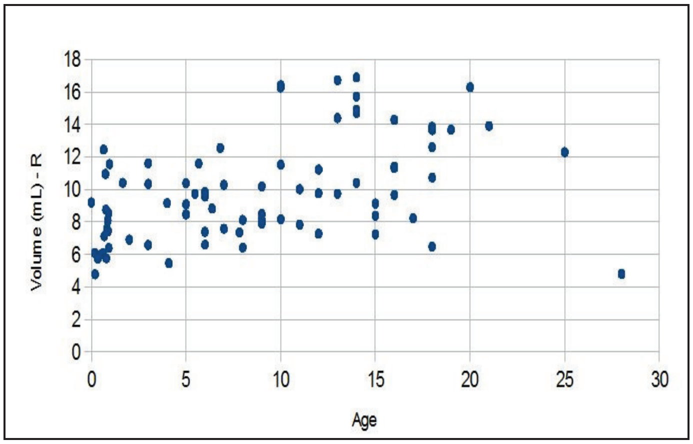

Right Volume

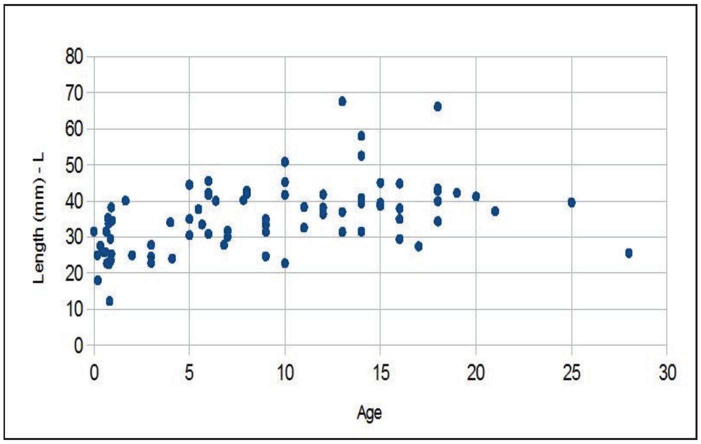

Left Length

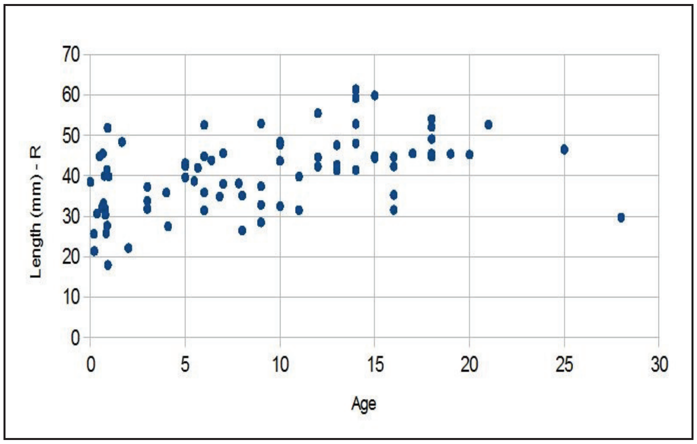

Right Length

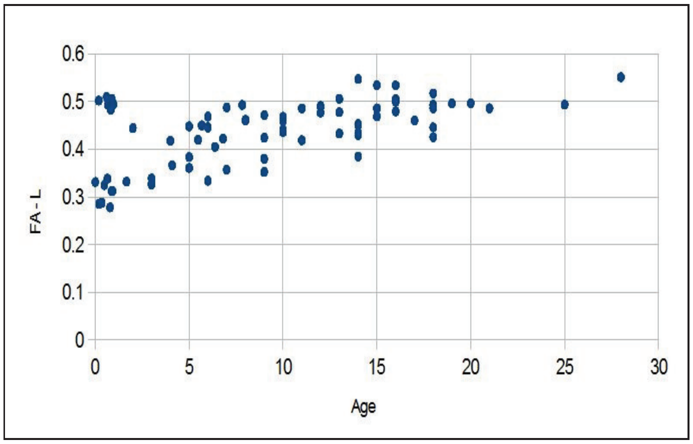

Left FA

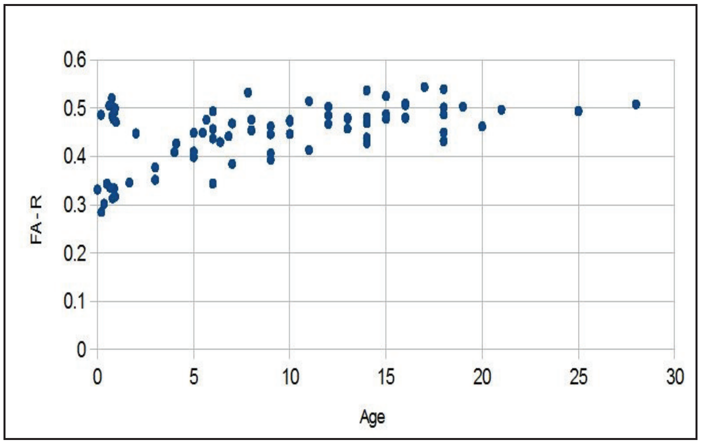

Right FA

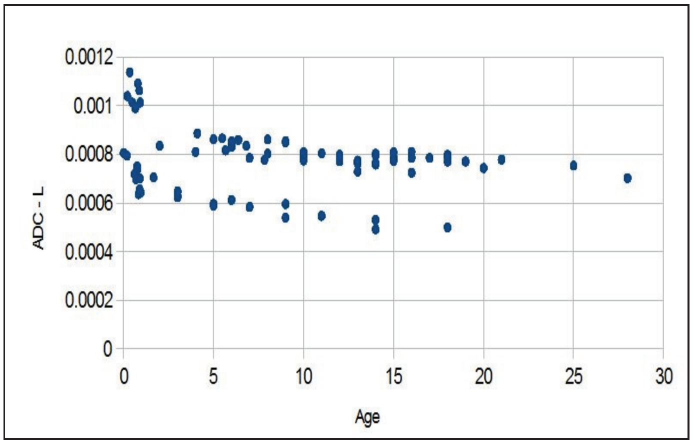

Left ADC

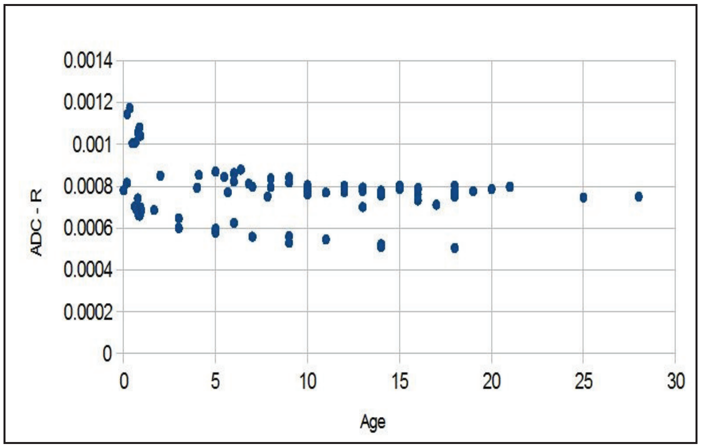

Right ADC

Posterior Arcuate - Raw Data

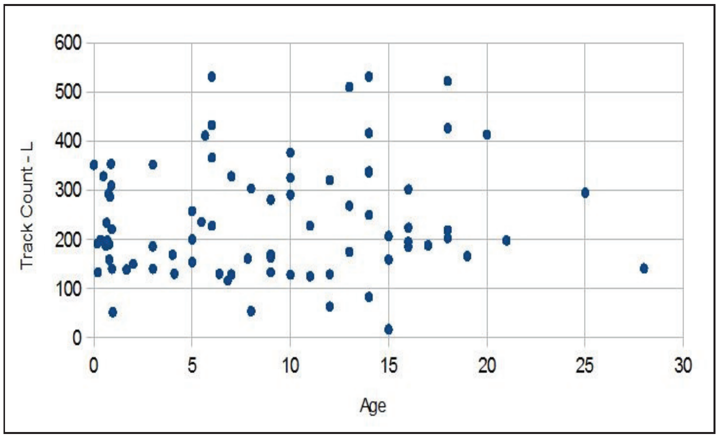

Left Track Count

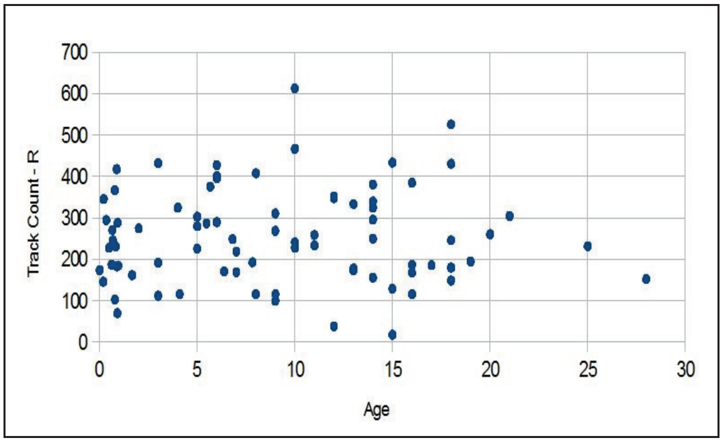

Right Track Count

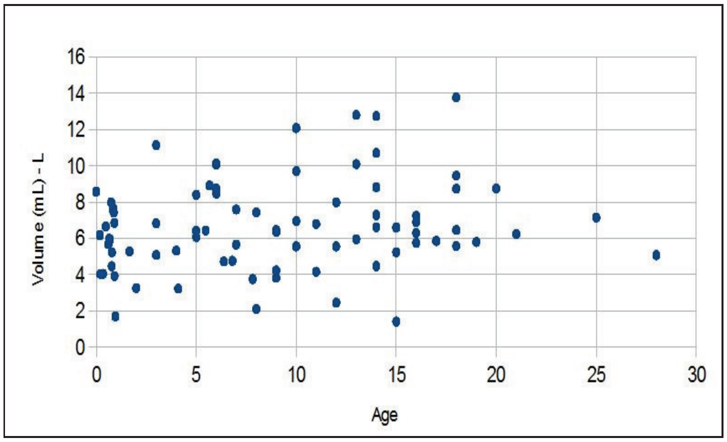

Left Volume

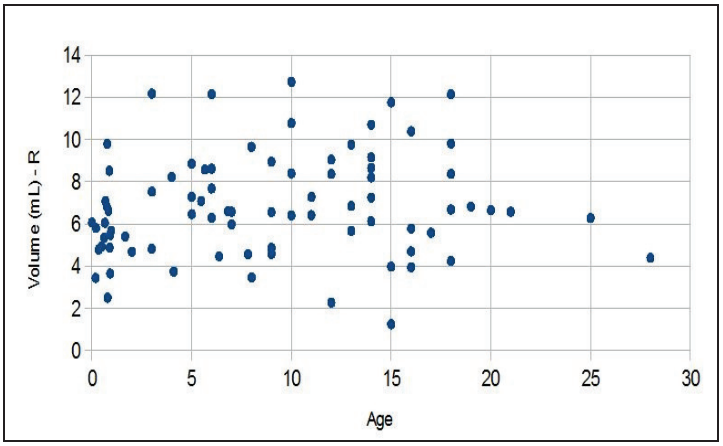

Right Volume

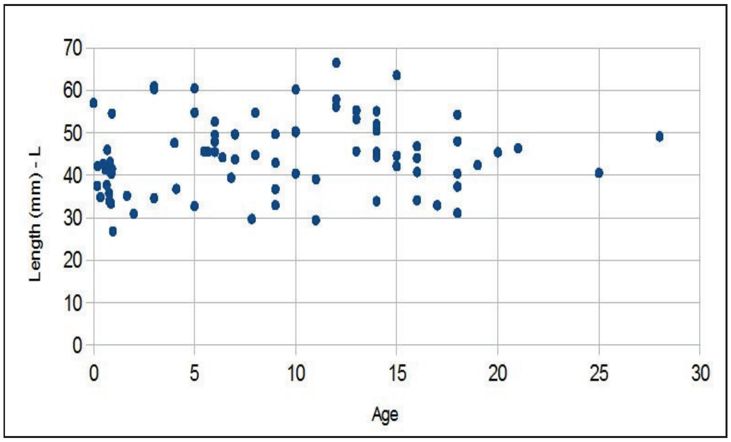

Left Length

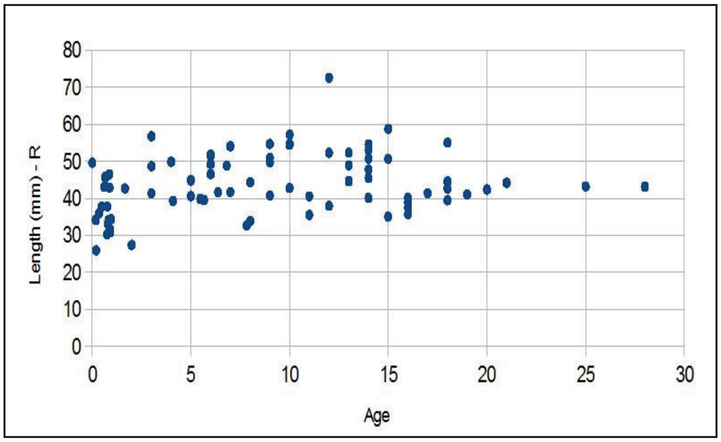

Right Length

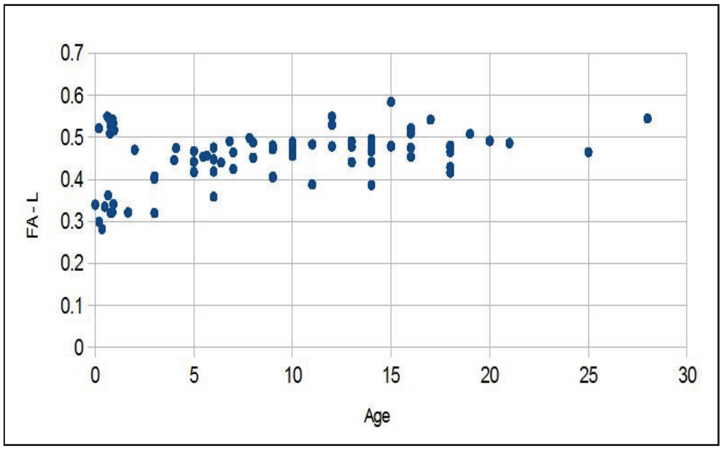

Left FA

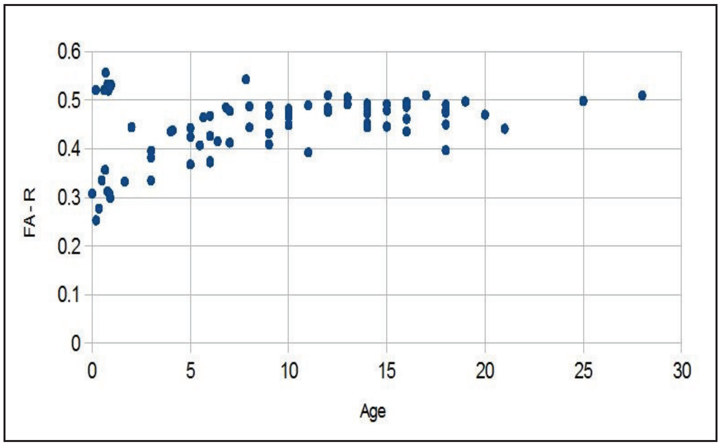

Right FA

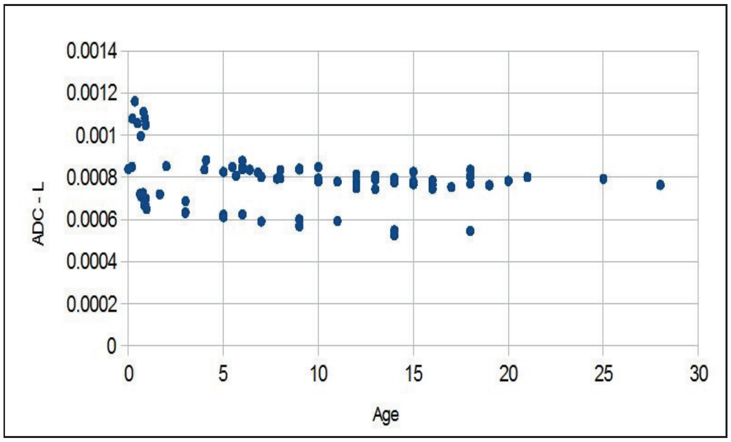

Left ADC

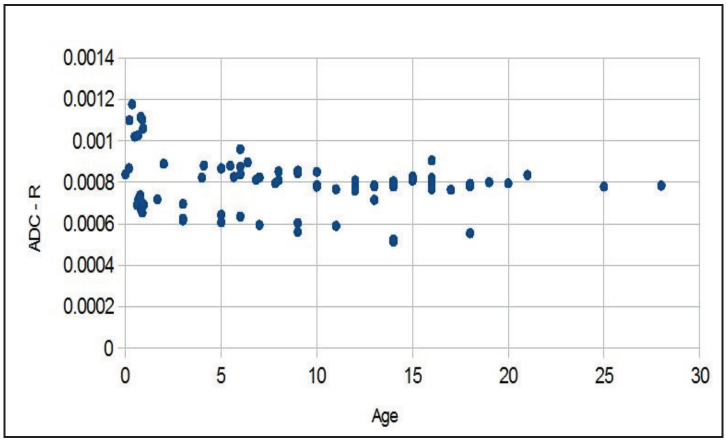

Right ADC

Long Arcuate - Raw Data

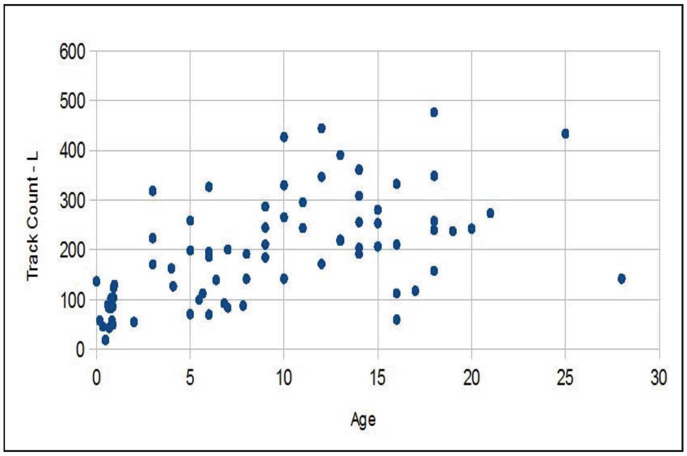

Left Track Count

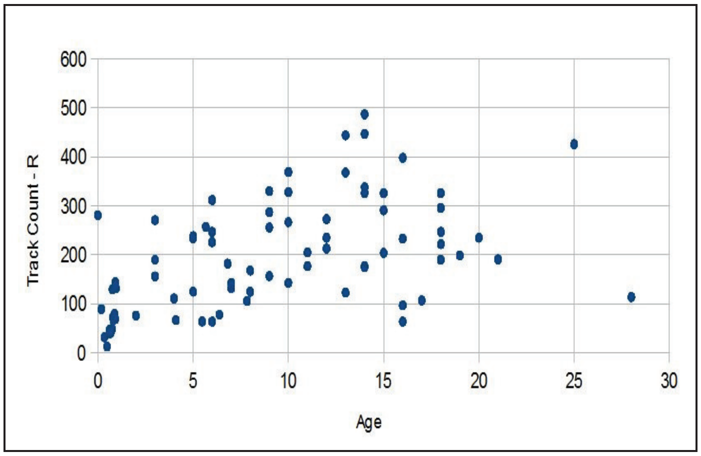

Right Track Count

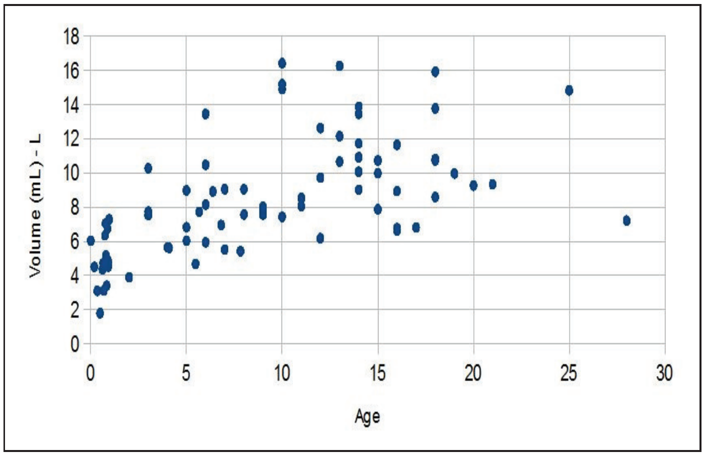

Left Volume

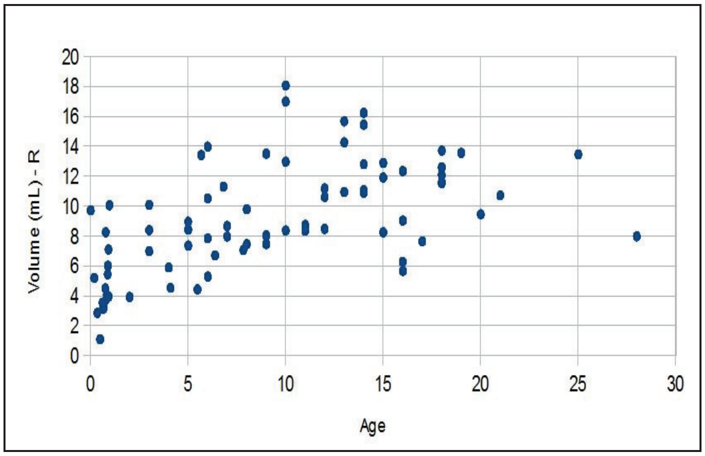

Right Volume

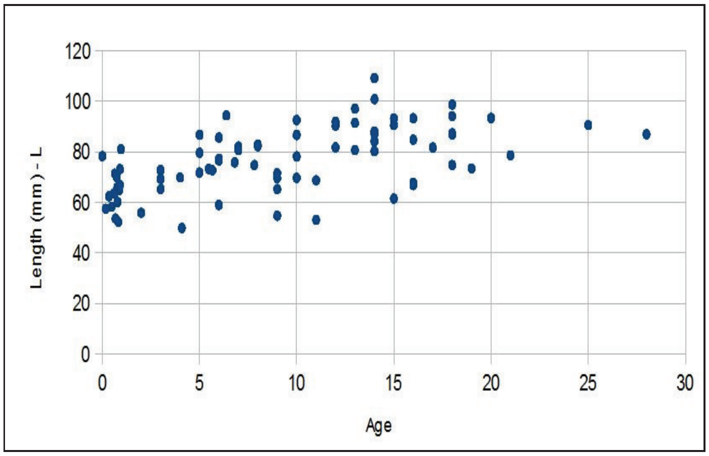

Left Length

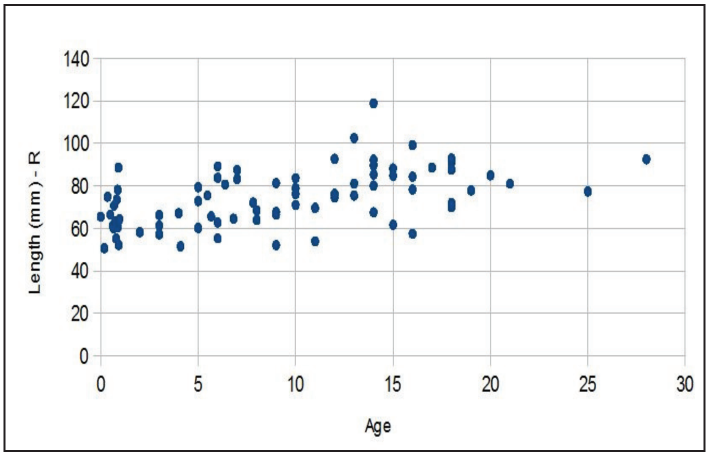

Right Length

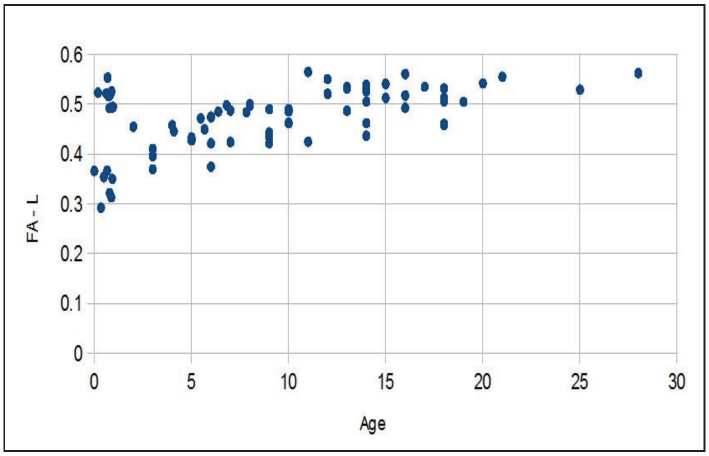

Left FA

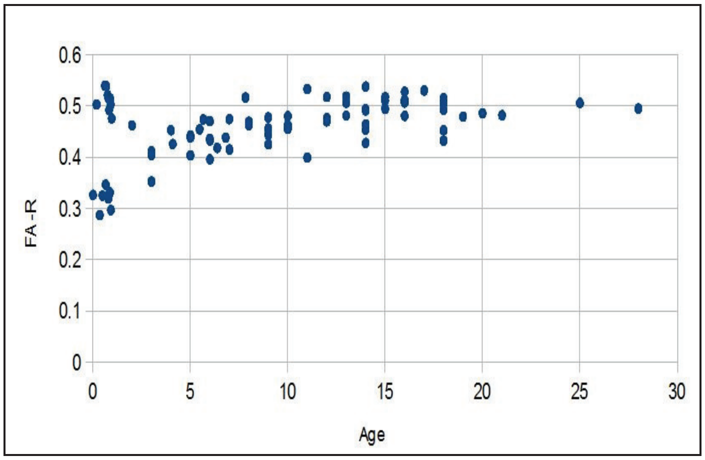

Right FA

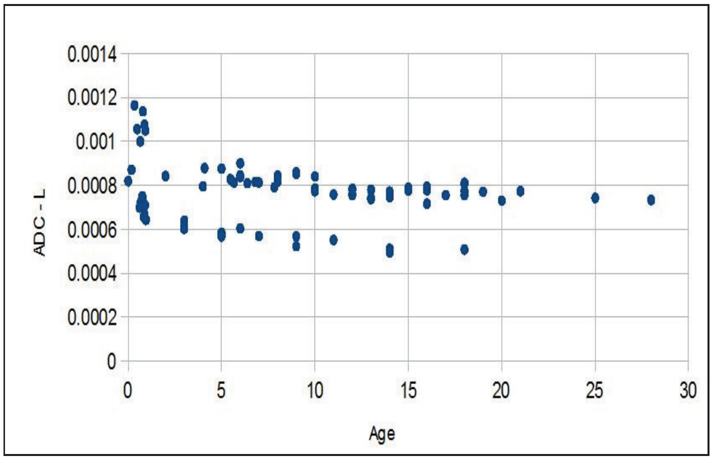

Left ADC

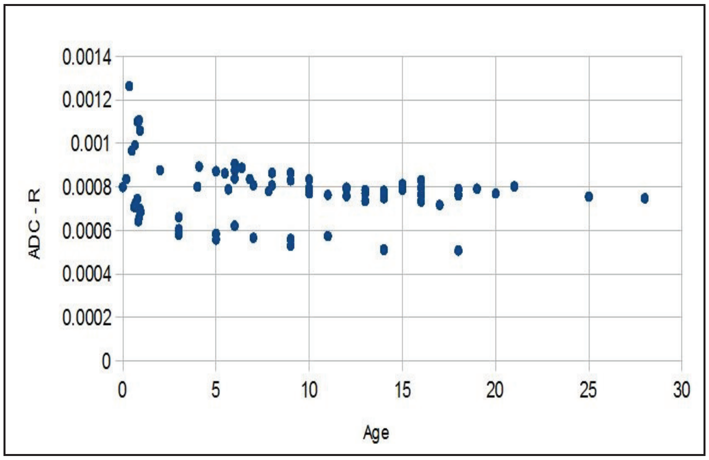

Right ADC
