# Supplementary material for: Detection and Growth Pattern of Arcuate Fasciculus from Newborn to Adult
Source: Front Neurosci. 2017 Jul 14;11:389. doi: 10.3389/fnins.2017.00389 (PMC5509799; doi:10.3389/fnins.2017.00389)
Supplement: Supplementary file 7 [file Image7.PDF]

# Anterior Arcuate - Whole Brain Normalization

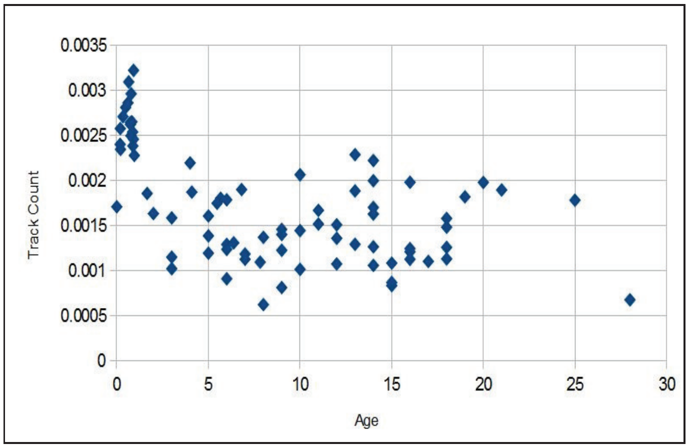


Track Count

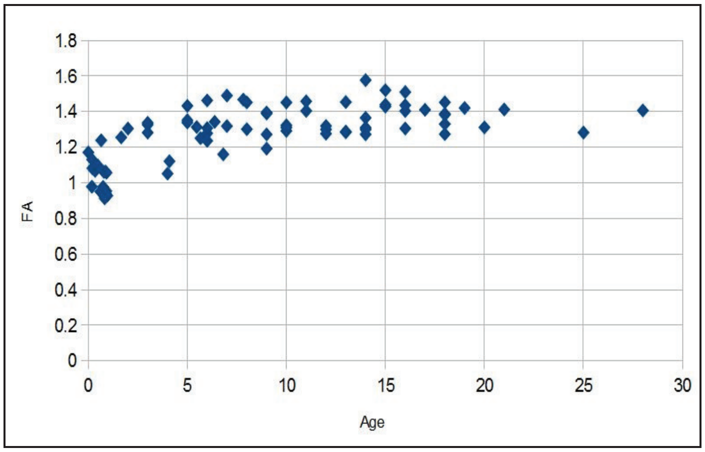


FA

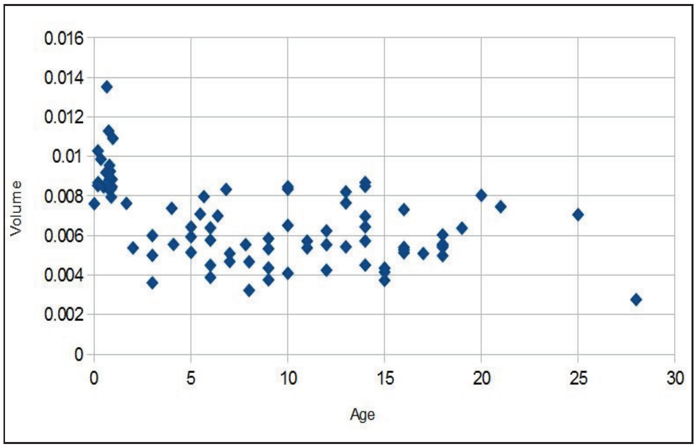


Volume

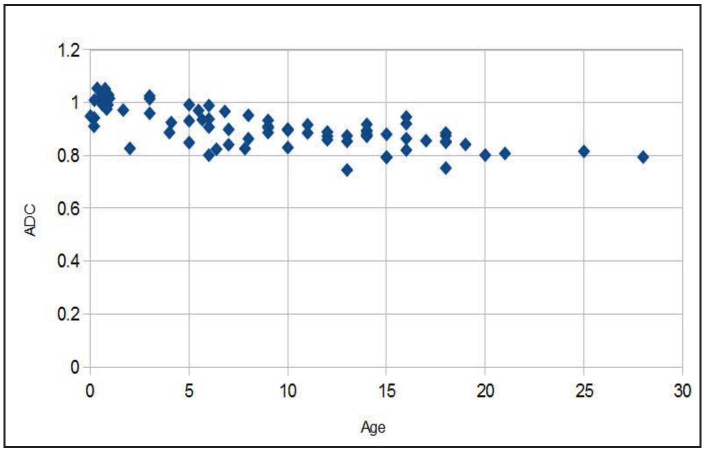


ADC

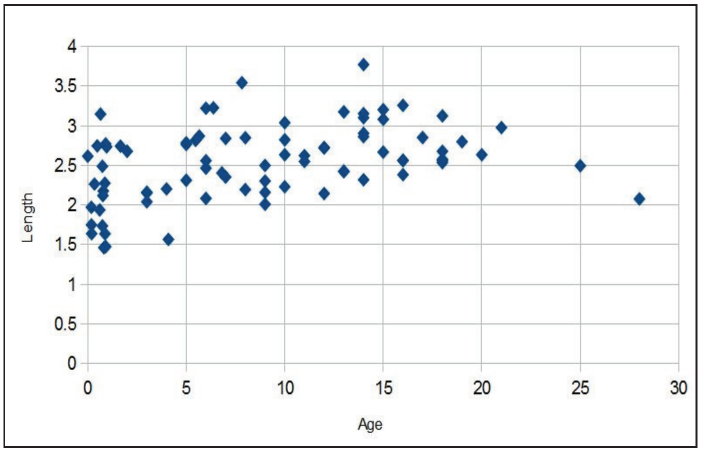


Length

## Posterior Arcuate - Whole Brain Normalization

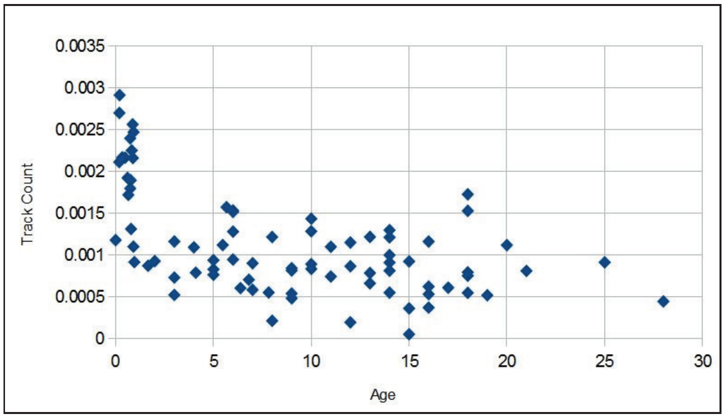


Track Count

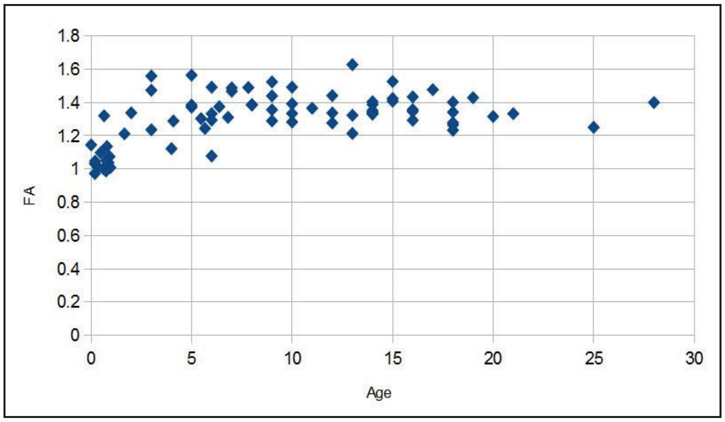


FA

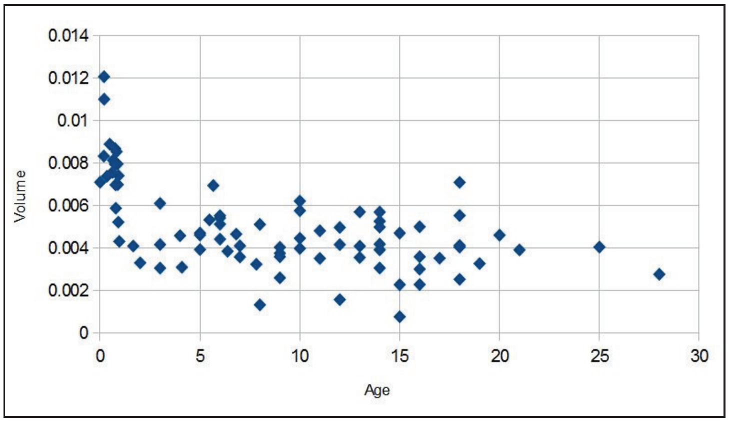


Volume

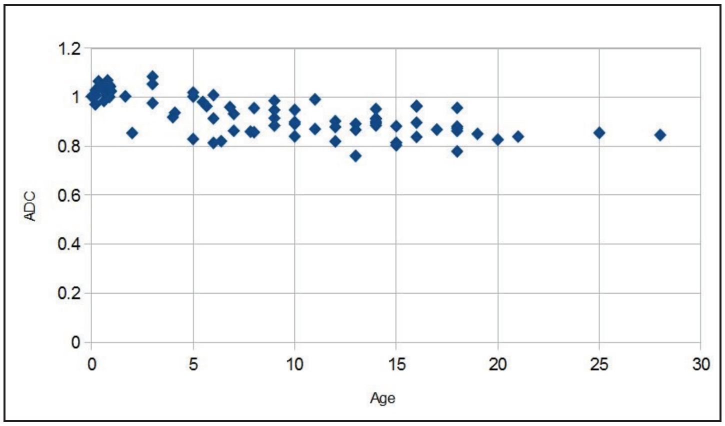


ADC

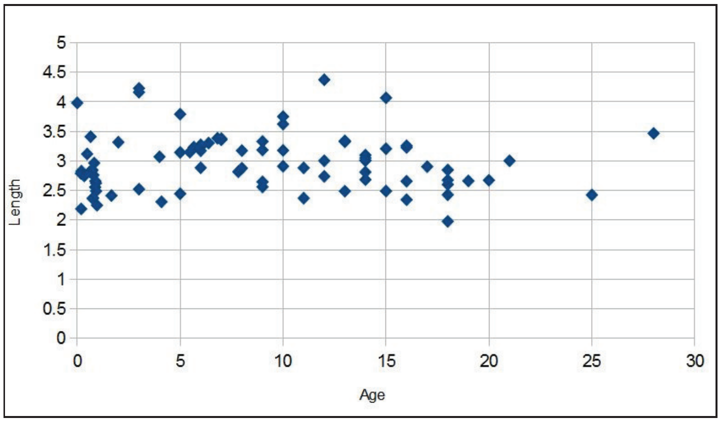


Length

## Long Arcuate - Whole Brain Normalization

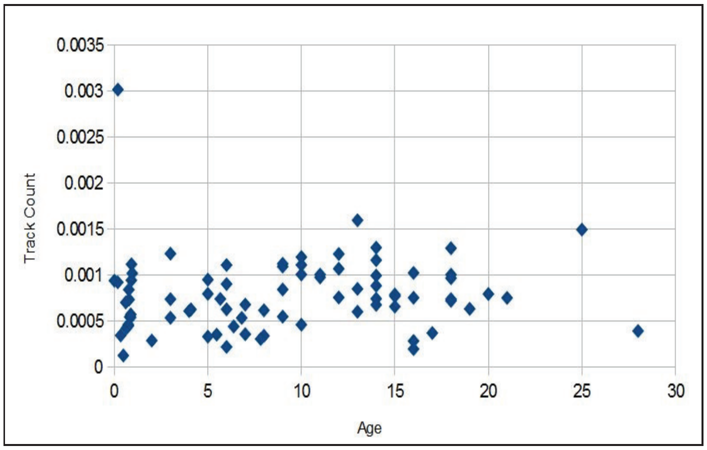


Track Count

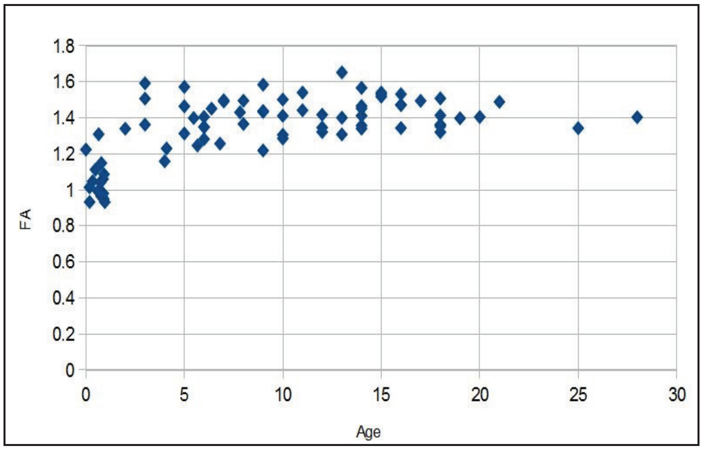


FA

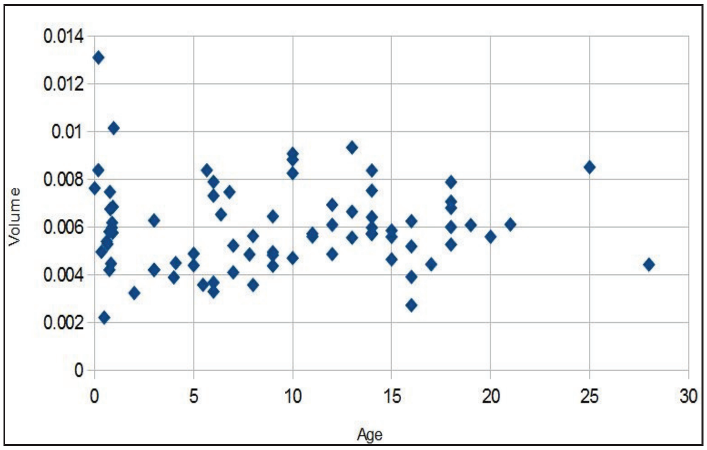


Volume

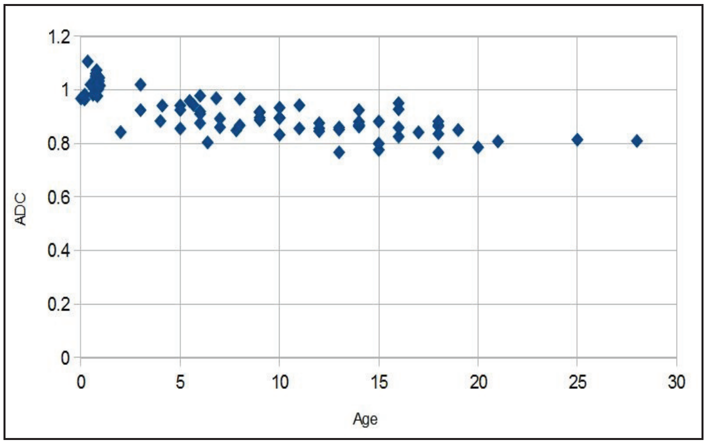


ADC

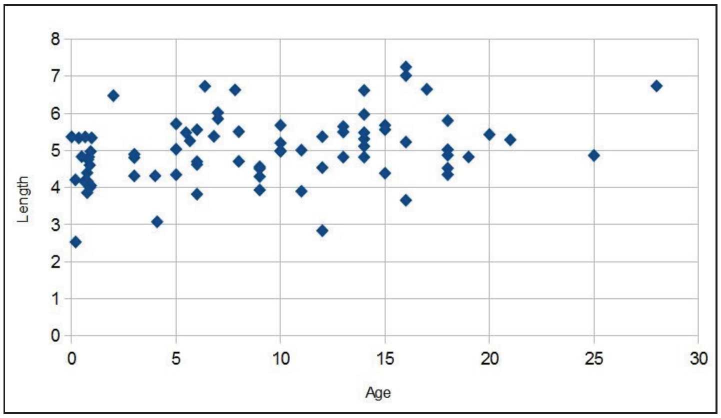


Length
