# Supplementary material for: Detection and Growth Pattern of Arcuate Fasciculus from Newborn to Adult
Source: Front Neurosci. 2017 Jul 14;11:389. doi: 10.3389/fnins.2017.00389 (PMC5509799; doi:10.3389/fnins.2017.00389)
Supplement: Supplementary file 8 [file Image8.PDF]

Anterior Arcuate - Laterality

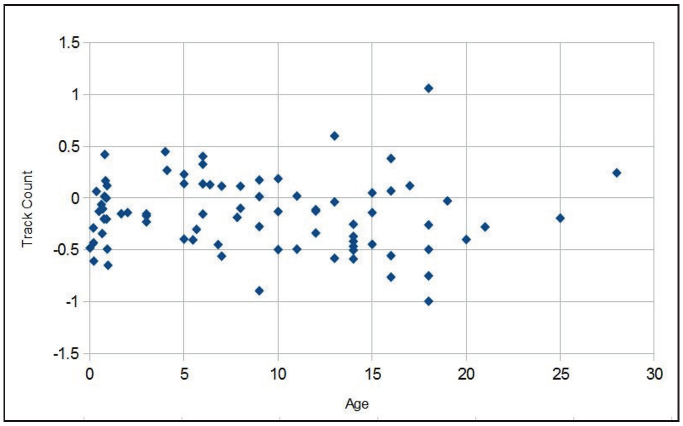

Track Count

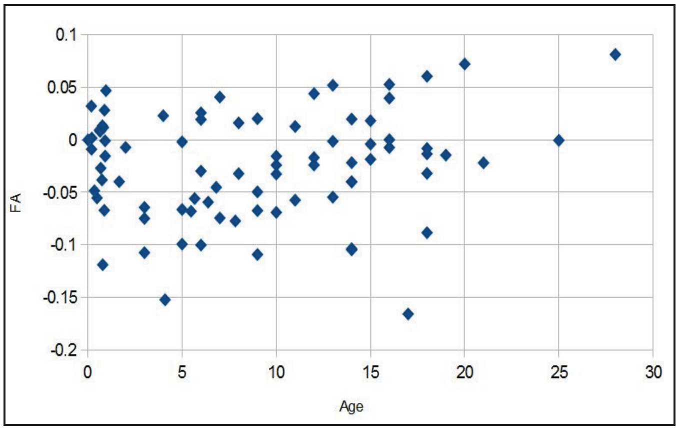

FA

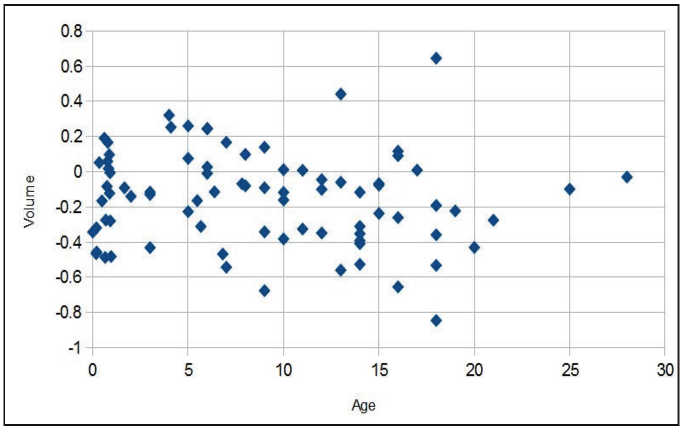

Volume

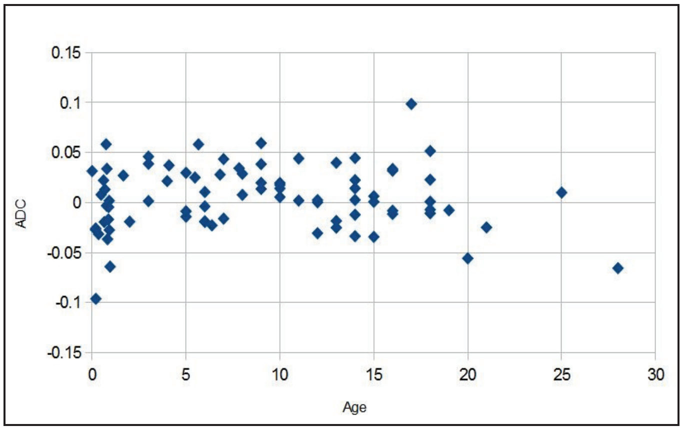

ADC

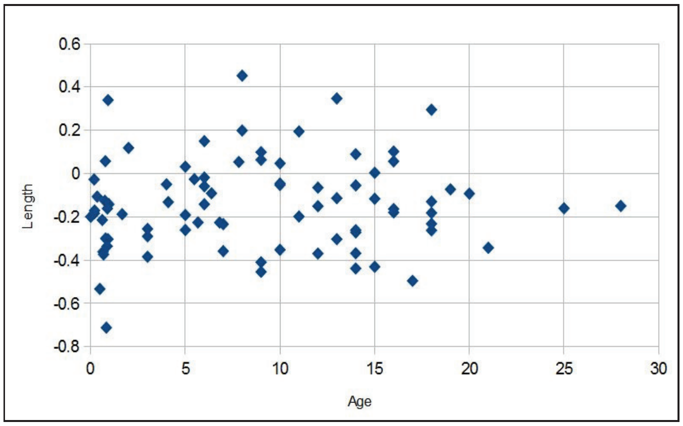

Length

Posterior Arcuate - Laterality

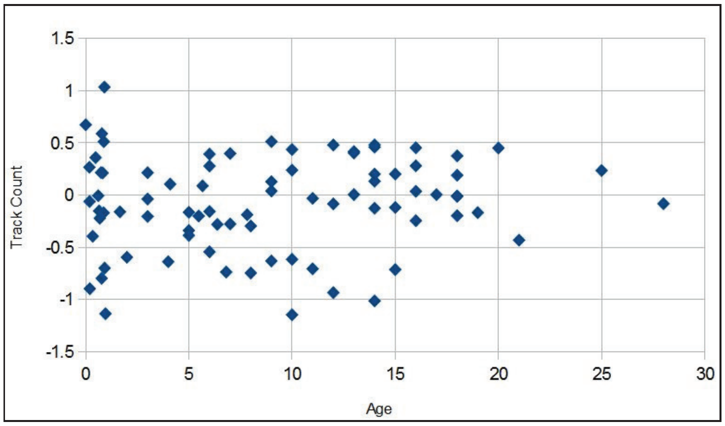

Track Count

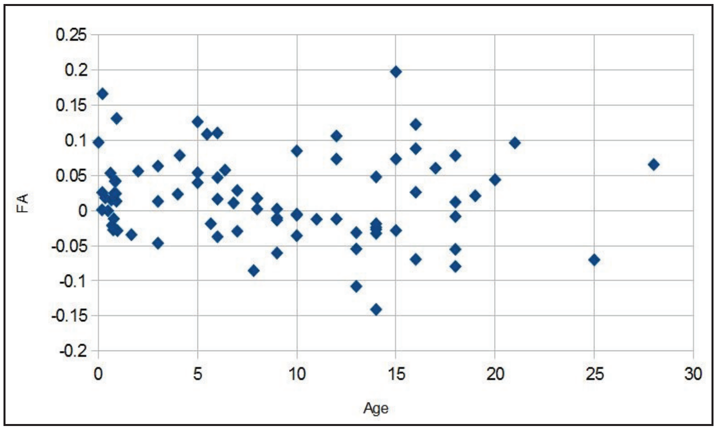

FA

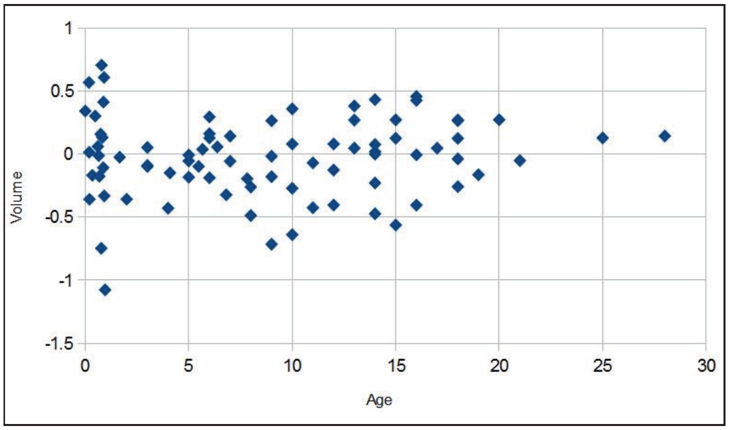

Volume

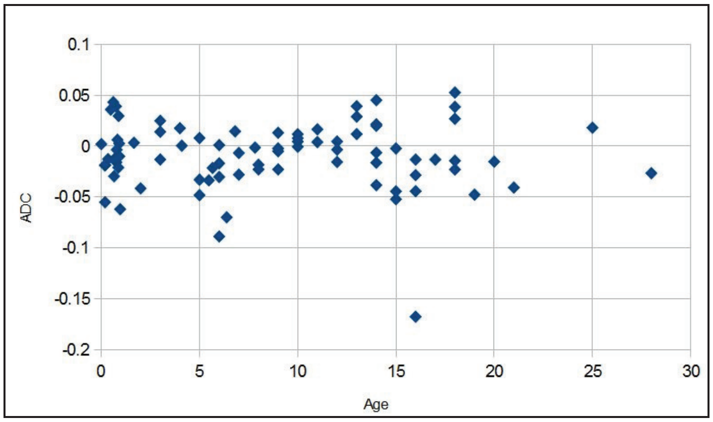

ADC

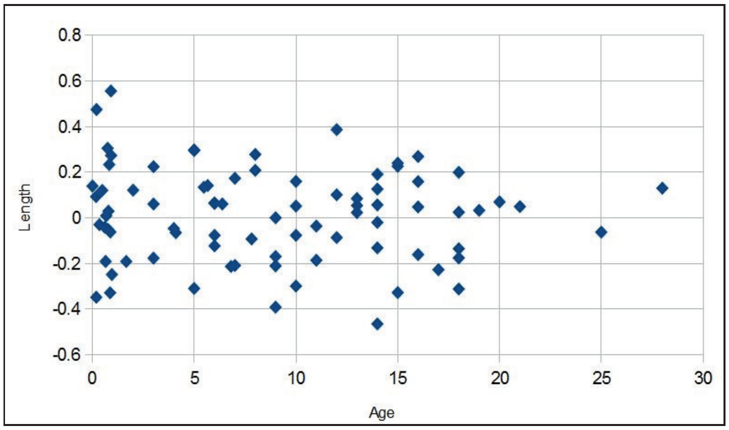

Length

Long Arcuate - Laterality

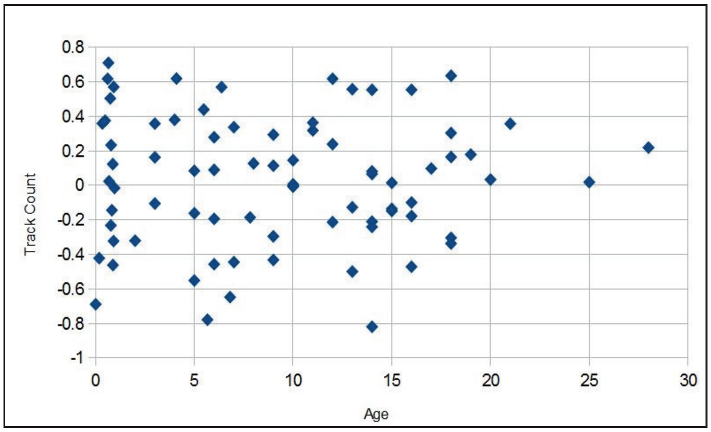

Track Count

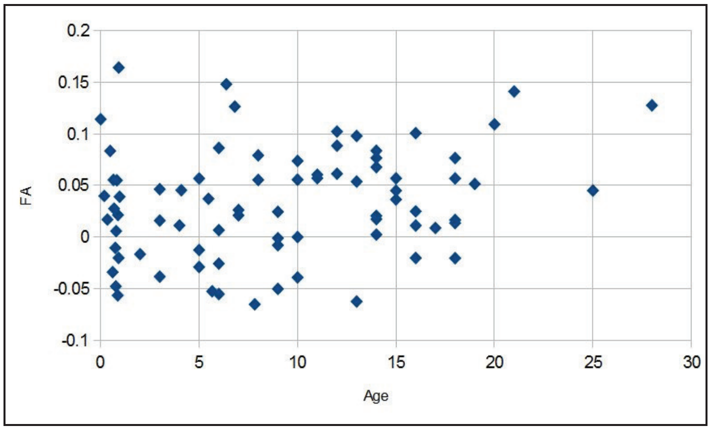

FA

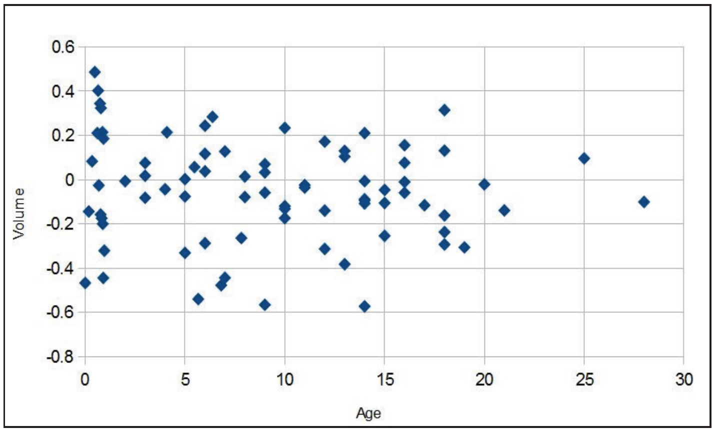

Volume

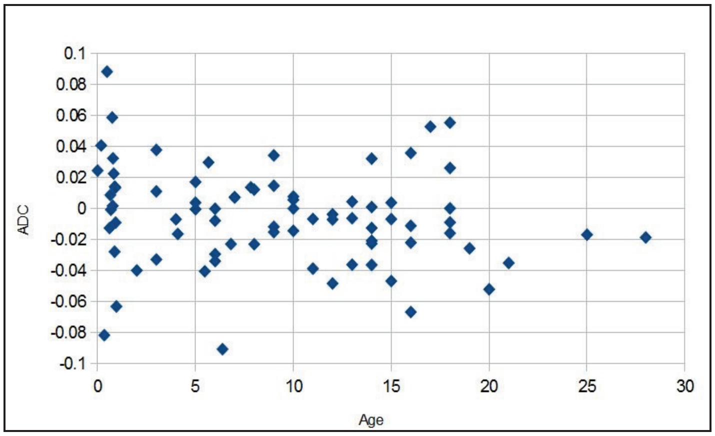

ADC

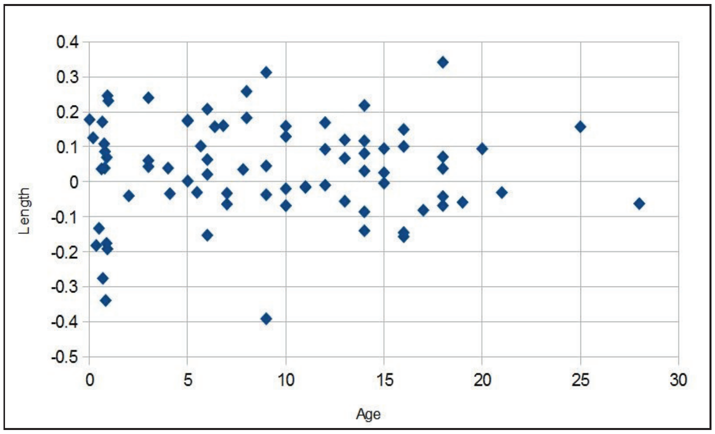

Length
